# A Virtual Infection Model Quantifies Innate Effector Mechanisms and *Candida albicans* Immune Escape in Human Blood

**DOI:** 10.1371/journal.pcbi.1003479

**Published:** 2014-02-20

**Authors:** Kerstin Hünniger, Teresa Lehnert, Kristin Bieber, Ronny Martin, Marc Thilo Figge, Oliver Kurzai

**Affiliations:** 1 Septomics Research Center, Friedrich Schiller University and Leibniz Institute for Natural Product Research and Infection Biology – Hans-Knöll-Institute (HKI), Jena, Germany; 2 Applied Systems Biology, Leibniz Institute for Natural Product Research and Infection Biology – Hans-Knöll-Institute (HKI), Jena, Germany; 3 Friedrich Schiller University Jena, Jena, Germany; Utrecht University, Netherlands

## Abstract

*Candida albicans* bloodstream infection is increasingly frequent and can result in disseminated candidiasis associated with high mortality rates. To analyze the innate immune response against *C. albicans*, fungal cells were added to human whole-blood samples. After inoculation, *C. albicans* started to filament and predominantly associate with neutrophils, whereas only a minority of fungal cells became attached to monocytes. While many parameters of host-pathogen interaction were accessible to direct experimental quantification in the whole-blood infection assay, others were not. To overcome these limitations, we generated a virtual infection model that allowed detailed and quantitative predictions on the dynamics of host-pathogen interaction. Experimental time-resolved data were simulated using a state-based modeling approach combined with the Monte Carlo method of simulated annealing to obtain quantitative predictions on *a priori* unknown transition rates and to identify the main axis of antifungal immunity. Results clearly demonstrated a predominant role of neutrophils, mediated by phagocytosis and intracellular killing as well as the release of antifungal effector molecules upon activation, resulting in extracellular fungicidal activity. Both mechanisms together account for almost 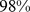 of *C. albicans* killing, clearly proving that beside being present in larger numbers than other leukocytes, neutrophils functionally dominate the immune response against *C. albicans* in human blood. A fraction of *C. albicans* cells escaped phagocytosis and remained extracellular and viable for up to four hours. This immune escape was independent of filamentation and fungal activity and not linked to exhaustion or inactivation of innate immune cells. The occurrence of *C. albicans* cells being resistant against phagocytosis may account for the high proportion of dissemination in *C. albicans* bloodstream infection. Taken together, iterative experiment–model–experiment cycles allowed quantitative analyses of the interplay between host and pathogen in a complex environment like human blood.

## Author Summary

*Candida albicans* is the most important fungal pathogen in nosocomial bloodstream infections. So far little is known about the interplay of different cellular and non-cellular immune mechanisms mediating the protective response against *C. albicans* in blood. The *in vivo* scenario of *C. albicans* infection can be mimicked by human whole-blood infection assays to analyze the innate immune response against this pathogen. These experiments reveal the time-evolution of certain mechanisms while leaving the values of other quantities in the dark. To shed light on quantities that are not experimentally accessible, we exploited the descriptive and predictive power of mathematical models to estimate these parameters. The combination of experiment and theory enabled us to identify and quantify the main course of the immune response against *C. albicans* in human blood. We quantified the central role of neutrophils in the defence against this fungal pathogen, both directly by phagocytosis and indirectly by secreting antimicrobial factors inducing extracellular killing. Other findings include the distribution of *C. albicans* cells in neutrophils and monocytes as well as the immune escape of *C. albicans* cells in the course of infection.

## Introduction

Sepsis is a systemic inflammatory response triggered by infection and a major cause of death worldwide [1]–[3]. In recent years, fungal pathogens have caused an increasing number of sepsis cases with high mortality rates [4], [5]. The major fungal pathogen *Candida albicans* is a common human commensal but can become invasive in patients with a compromised immune system and disturbance of epithelial barrier integrity or may enter the bloodstream by disseminating from biofilms on medical devices [6]–[8]. Among the different components of human immunity, neutrophils (polymorphonuclear neutrophilic granulocytes, PMN) are crucial for antifungal immune responses and neutropenia is associated with impaired prognosis in systemic candidiasis [9]. PMN possess several mechanisms that may contribute to clearing of *C. albicans* like phagocytosis, oxidative burst, degranulation and formation of neutrophil extracellular traps (NETs) and have been shown to respond specifically to the invasive filamentous form of *C. albicans* [10]. Other peripheral blood immune cells have also been implicated in the response against *C. albicans*, including monocytes as well as NK-cells [11], [12]. Furthermore, *C. albicans* has been shown to strongly activate complement while at the same time recruiting complement regulators to its surface that may protect it against antimicrobial effector functions [13]–[17]. So far little is known about the interplay of these effects *in vivo*. Studies using purified human immune cells or experiments performed at a molecular level provide important insights into mechanisms of immune recognition but fail to address *in vivo* complexity. Murine models are mainly used to address *in vivo* settings but peripheral blood components differ substantially from their human counterparts with regard to quantity and functional aspects [18]. To overcome some of these limitations, a human whole-blood infection model can be used to monitor host-pathogen interactions. Such models have successfully been used in identifying microbial virulence factors [19], analyzing early immune responses [20], determining the influence of genetic polymorphisms on immune response [21] and testing potential therapeutic approaches or vaccine efficacy [22]–[26]. Whole-blood assays provide time-resolved data on localization and physiological state of the pathogen and immune activation. Whereas many parameters are accessible to direct experimental quantification, others are not due to experimental limitations. However, biomathematical modeling can provide tools to overcome these experimental limitations. Here, we formulate a mathematical infection model for *C. albicans* in human blood and apply a state-based modeling approach to perform computer simulations that predict details on the dynamics of the immune response. The state-based model corresponds to a non-spatial agent-based model that enables decision making depending on the occurrence of specific events, such as first-time phagocytosis, and allows modeling interactions between individual cells occurring in small numbers in a stochastic fashion [27]. We demonstrate that *a priori* unknown transition rates between any two states can be estimated by fitting the simulation results to the experimental data using the Monte Carlo method of simulated annealing. Therefore, the state-based model allows detailed predictions on dynamics of host-pathogen interaction in human blood and, in particular, on the main course of the immune response.

## Results

### *C. albicans* induces a strong pro-inflammatory response in human blood

**Figure 1:**
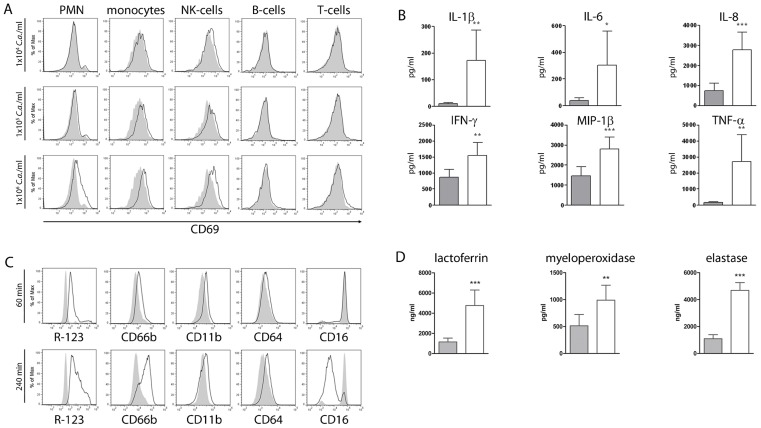
*C. albicans* infection of human whole blood results in rapid activation of cellular innate immunity. (A) Human whole blood was infected with different amounts of *C. albicans* yeasts for 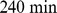 and changes in CD69 surface levels (black open histograms) on immune cells were analyzed. Filled grey histograms indicate basal CD69 expression. The early activation marker CD69 was increased on PMN, monocytes and NK-cells following inoculation of 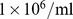 *C. albicans*. In contrast, no changes in CD69 surface levels could be observed on T-cells and B-cells. Inoculation of whole blood with lower amounts of *C. albicans* resulted in less efficient immune activation. Data from one of three independent experiments using cells from different donors with virtually identical results are shown. (B) Significantly increased plasma levels of pro-inflammatory cytokines (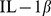, IL-6, 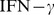, 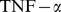) as well as chemokines (IL-8, 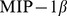) could be detected after 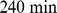 of infection with *C. albicans* (white bars). Grey bars indicate basal plasma levels in mock-infected samples. Bars show means 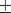 standard deviation of at least 4 independent experiments with whole blood from different donors, 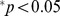, 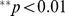, 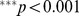. (C) PMN activation is shown 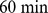 (upper panel) and 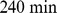 (lower panel) after inoculation of *C. albicans* into whole blood by detection of intracellular generated reactive oxygen intermediates (oxidation of dihydrorhodamine-123 to rhodamine-123, R-123) and by changes in the surface expression levels of activation markers CD66b, CD11b, CD64 (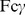 receptor I) and CD16 (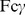 receptor III). Grey filled histograms indicate basal expression on PMN from mock-infected samples, black open histograms indicate surface levels following *C. albicans* inoculation. Data from one of at least three independent experiments with virtually identical results are shown. (D) Plasma samples of whole-blood infection experiments were analyzed for the release of myeloperoxidase, lactoferrin and elastase from neutrophil granules. Grey bars show the basal levels within mock-infected blood, white bars show levels after inoculation with *C. albicans* (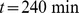). The release of the three antimicrobial peptides was significantly enhanced after contact to the fungus. Bars show means 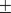 standard deviation of at least 4 independent experiments with whole blood from different donors, 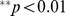, 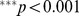.

To analyze early immune responses to a fungal pathogen, *C. albicans* was added to lepirudin-anticoagulated whole-blood of healthy volunteers at different concentrations. After inoculation of 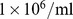 *C. albicans* yeasts, activation of PMN, monocytes and NK-cells but no unspecific early activation of T- and B-cells could be detected by quantification of the general activation marker CD69 (Fig. 1A). Furthermore, no cell death or decrease in host cell numbers was observed with this inoculum throughout the course of the experiment. No or only slight changes in CD69 expression levels could be observed in response to lower concentrations of *C. albicans* (Fig. 1A). Fungal concentrations of 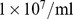 and more resulted in significant host cell death at later stages of infection. Therefore an inoculum of 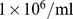 *C. albicans* yeasts was used in subsequent experiments. Innate immune activation by *C. albicans* resulted in significantly elevated plasma levels of pro-inflammatory cytokines (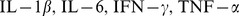) as well as chemokines (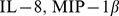) (Fig. 1B). As PMN have been shown to play a central role in the defense against *C. albicans*, we quantified activation of these cells in more detail. Early after inoculation of *C. albicans* a strong induction of reactive oxygen intermediates in PMN could be observed (Fig. 1C). Surface levels of receptors involved in immune recognition like CD11b and CD64 increased, whereas CD16 markedly decreased on PMN after fungal inoculation indicating cellular activation (Fig. 1C). Up-regulated surface exposure of the degranulation marker CD66b and increased plasma concentrations of myeloperoxidase, lactoferrin and elastase confirmed massive degranulation (Fig. 1D). Consequently, activation of neutrophils also resulted in the accumulation of potentially fungicidal activity in plasma [28], [29].

### *C. albicans* associates with PMN in human blood

**Figure 2:**
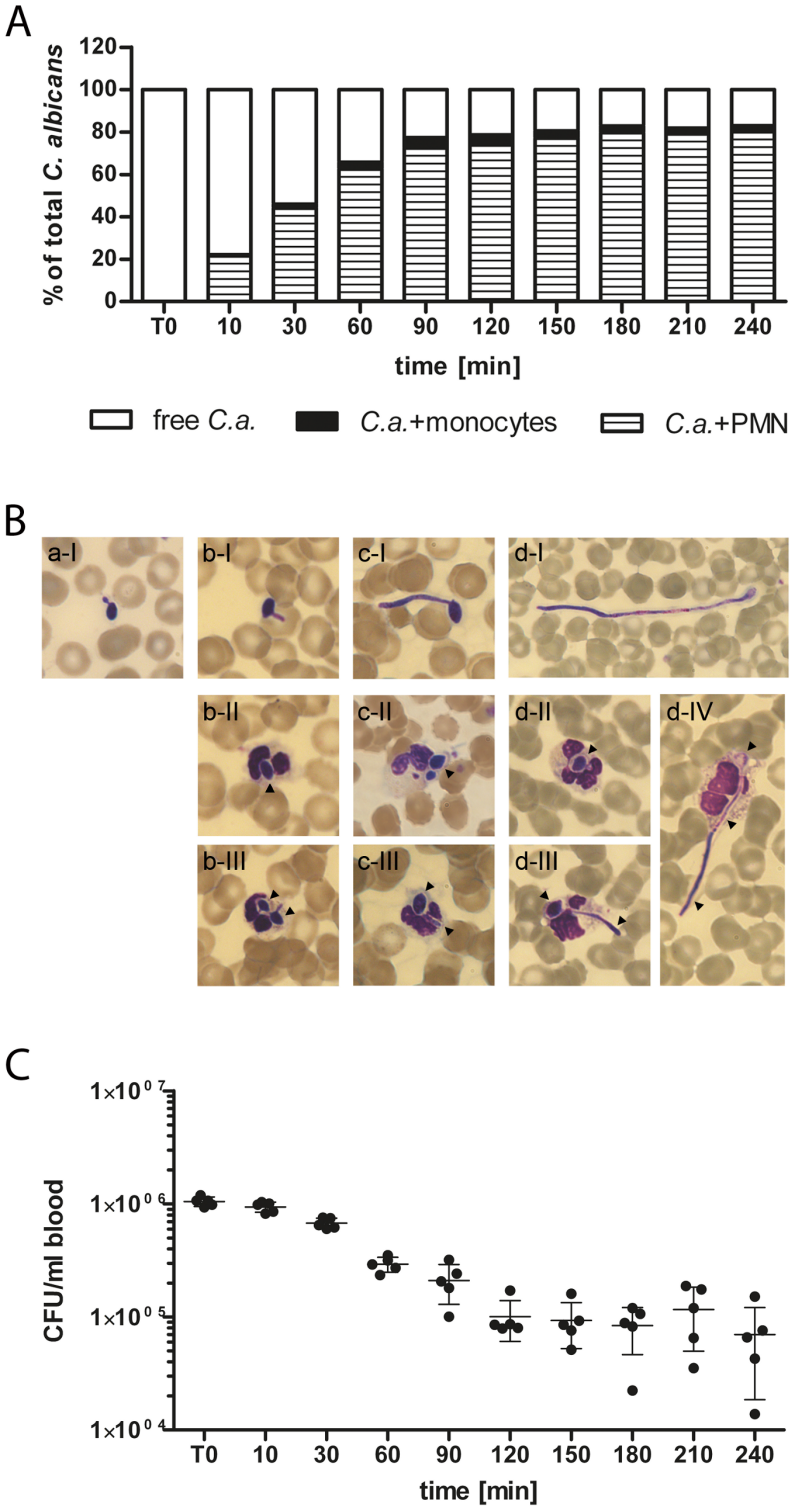
*C. albicans* predominantly associates with PMN and is killed rapidly. (A) Time-dependent increase of *C. albicans* association with blood cells as determined by flow cytometry. The majority of *C. albicans* cells associated to PMN whereas only low interactions could be observed for monocytes and no association to lymphocytes was detectable. The percentages of *C. albicans* associated with PMN (striped bars) or monocytes (black bars) were calculated relative to total *C. albicans* cells in blood (set to 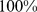). All values correspond to the means of five independent experiments with whole blood from five different donors. (B) Representative blood smears of *C. albicans*-infected blood after 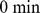 (a), 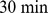 (b), 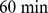 (c) and 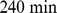 (d) demonstrate continuous filamentation of extracellular fungi (I). Ingested *C. albicans* (black arrows) were mainly found in PMN and showed different morphotypes. (C) Survival assay of *C. albicans* exposed to human whole blood shows a rapid killing of the fungus within 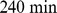 of infection. Each dot represents *C. albicans* colony forming units (*C. a.* CFU/ml blood) of independent experiments with blood from different donors. The mean 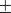 standard deviation is given for each time point.

To analyze the distribution of the fungal pathogen in different compartments of human blood we used a *C. albicans* strain constitutively expressing GFP. Within 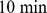 of blood infection 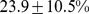 of fungal cells associated with PMN and this interaction was further increased at 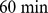 (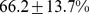) and 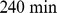 (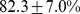). Whereas low association of *C. albicans* to monocytes (maximum association to monocytes at 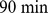 *p.i.* 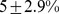) could be observed, no interactions with lymphocytes were detectable (Fig. 2A). A significant proportion of *C. albicans* cells (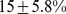 at 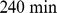) remained extracellularly throughout the observation period and therefore escaped the cellular immune response by developing resistance against phagocytosis. The inoculation of human blood with 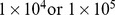 *C. albicans* yeasts/ml resulted in similar fungal association patterns indicating that distribution of *C. albicans* in blood is largely independent of the fungus to immune cell ratio. To test, whether this distribution pattern was characteristic for *C. albicans* or rather strain specific, we used a set of ten clinical isolates from bloodstream infections. All strains showed similar distribution patterns with a strongly predominant association to PMN (at 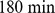 *p.i.* median association to PMN: 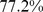 [range 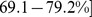, median association to monocytes: 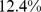 [range 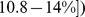. For none of the strains, association to lymphocytes could be detected. Concomitant to interaction with immune cells, changes in *C. albicans* morphology could be observed in microscopic analyses (Fig. 2B). Intracellular organisms were predominantly found in PMN throughout the experiment and showed different morphotypes, in line with a growth arrest of filaments in PMN after phagocytosis [10]. In contrast, extracellular fungi showed small germ tubes 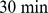 after inoculation and mainly occurred as pseudohyphae at later time points, indicating continuous filamentation of these cells during the experiment (Fig. 2B). Plating assays demonstrated a substantial killing of *C. albicans* over time with only 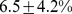 of fungal cells remaining viable four hours after inoculation (Fig. 2C).

### Virtual infection model quantifies mechanisms of the immune response

**Figure 3:**
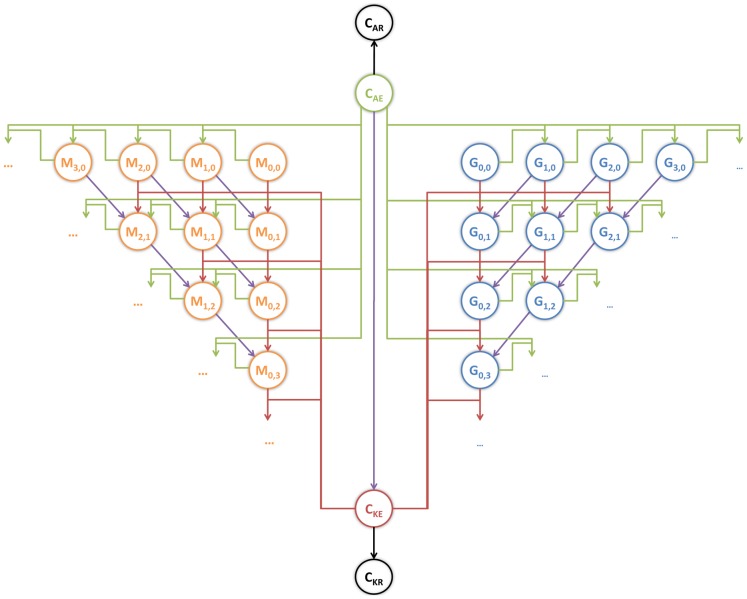
Schematic representation of the state-based model. Circular symbols depict different states of the model, *i.e.* the green circle represents extracellular alive *C. albicans* (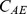), the red circle indicates extracellularly killed *C. albicans* (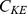), the black circles symbolize resistant *C. albicans* that are alive (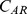) or killed (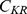), orange circles represent states of monocytes (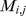) with 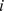 alive and 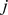 killed *C. albicans* and the blue circles depict different states of PMN (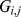). The model is not restricted by the number of immune cell states, as indicated by the dots, but is extended to account for all required states. The arrows represent allowed transitions between states, where their different colors correspond to the state of *C. albicans* (alive or dead) and the type of transition that they can perform (phagocytosis, killing or resistance). Alive *C. albicans* can be phagocytosed (green arrows), killed (purple arrows) or can became resistant (black arrow). *C. albicans* that are already killed can be phagocytosed (red arrows).

**Figure 4:**
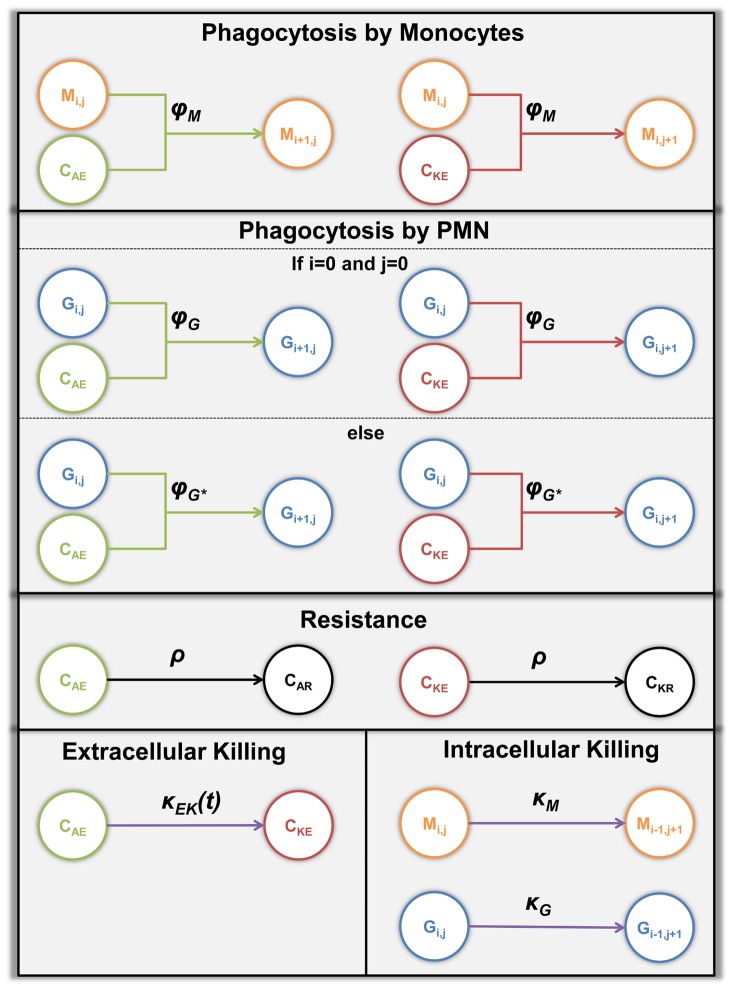
Types of transition in the state-based model. Illustration of all types of transitions, arranged according to their effect on *C. albicans*. Circular symbols depict different states and arrows represent transitions between states. Each transition type is associated with a specific transition rate. Extracellular alive *C. albicans* can be extracellularly killed by antimicrobial effector molecules, *i.e.* transition from state 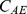 to 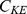, with rate 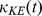. Alive as well as killed *C. albicans* can become resistant *C. albicans*, *i.e.* transition from state 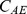 to state 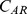 and transition from state 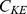 to state 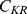, with transition rate 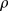. Furthermore, alive and dead extracellular *C. albicans* can be phagocytosed by monocytes with rate 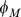 and by PMN that phagocytose for the first time or at least for the second time with rate 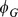 and 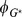, respectively. Alive *C. albicans* that were already phagocytosed can be killed intracellular in monocytes with transition rate 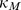 as well in PMN with rate 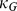. The monocytes and PMN containing 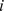 alive and 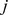 killed *C. albicans* are represented by 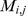 and 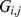, respectively.

To model host-pathogen interaction in *C. albicans* blood infection we used a state-based model that comprises all experimentally validated *C. albicans* states in human blood (Fig. 3, for details see Methods section and a flow-diagram of the algorithm in Fig. S1). Alive *C. albicans* cells (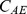) may be extracellularly killed (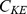) and both, 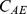 and 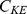 may turn into cells that are resistant against phagocytosis and further killing, denoted by 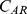 and 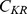, respectively. Non-resistant extracellular cells may be phagocytosed by monocytes or PMN and internalized viable fungal cells could be killed intracellularly. A proper bookkeeping of these intracellular processes in monocytes (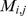) or granulocytes (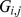) was ensured by the two indices, which refer to the numbers of internalized *C. albicans* cells that are alive (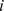) and killed (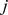), respectively. Transitions between states occur with specific transition rates that determine the time-dependent simulation of the infection process and are summarized in Fig. 4. Of note, we distinguished the initial phagocytosis by PMN with rate 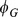 from subsequent phagocytosis events by activated PMN that may occur with a different rate 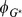 [30]. Furthermore, taking into account that the release of antimicrobial peptides by PMN induces extracellular killing, we used a time-dependent rate 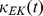 for extracellular killing that increases with the number of initial phagocytosis events by PMN.

**Figure 5:**
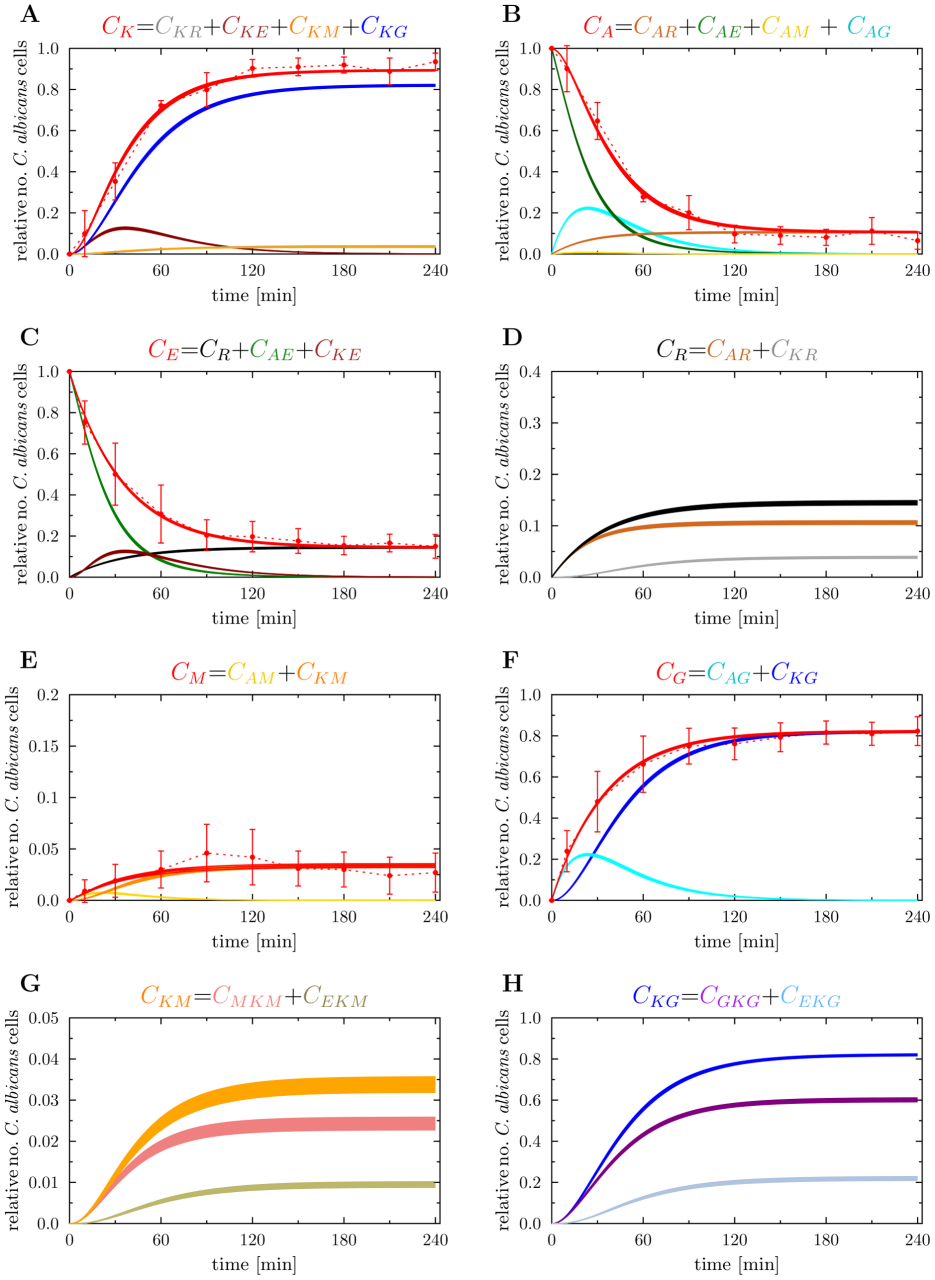
Result of the state-based model simulation generated by estimated transition rates. Time course of different combinations of simulated data (red solid lines) were fitted to associated experimental data from whole-blood infection assays (red dotted lines as guide for the eye) with corresponding standard deviations. The thickness of the solid lines represents the mean 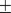 standard deviation of the simulation results that was obtained from 100 simulations for the normally distributed transition rates. Colored symbols refer to different *C. albicans* states, where their time courses are indicated by continuous lines with the same color. (A) Time-dependent relative number of killed *C. albicans* cells (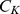) that were experimentally measured by survival plates. The experimental results were compared with the combination of simulated data representing all killed *C. albicans* of the model, *i.e.* extracellularly killed *C. albicans* (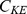), killed resistant *C. albicans* (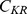), killed *C. albicans* that are in monocytes (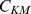) or PMN (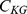). (B) Alive *C. albicans* (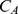) that were measured by survival plates and simulated by the combination of alive *C. albicans* that are in extracellular space (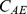), in monocytes (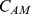), in PMN (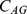) or became resistant against phagocytosis (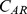). (C) Time course of *C. albicans* cells that are in extracellular space of blood (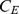). Experimental data was obtained by FACS analysis and simulated data is represented by the combination of *C. albicans* cells that are extracellular alive (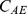), extracellularly killed (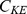) and resistant against phagocytosis (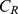). (D) The simulated resistant *C. albicans* (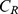) are the sum of alive and dead resistant *C. albicans* cells at each time point of the simulation time. (E) Time course of *C. albicans* cells that were phagocytosed by monocytes (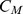). This is defined as sum of alive and killed *C. albicans* cells in monocytes, *i.e.* 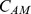 and 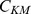, respectively. The corresponding experimental data was obtained by FACS analysis. (F) Relative number of *C. albicans* cells in PMN (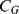) during the whole-blood infection, where internalized *C. albicans* cells can be alive (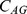) or dead (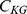). (G) Simulation result of killed *C. albicans* cells within monocytes (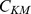), that is defined as the sum of internalized *C. albicans* that were intracellularly killed(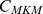) and those who were extracellularly killed (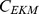). (H) Simulated time course of killed *C. albicans* cells in PMN (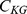), that is composed of intracellularly killed *C. albicans* cells (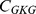) and extracellularly killed *C. albicans* cells (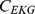) in PMN.

Initially, all immune cells occupied states 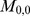 and 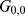 and the number of immune cells were set to average physiological numbers in blood: 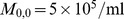 and 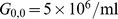. The initial number of *C. albicans* cells corresponded to the inocula used in the experiments and these cells were either in the 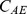-state or in the 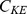-state, while no resistant cells existed at the initial time point. *A priori* unknown transition rates were estimated by the method of simulated annealing based on the Metropolis Monte Carlo Scheme. Starting with a randomly chosen parameter set, the algorithm searched in the parameter space of transition rates for the global optimum from a fit to the time-resolved experimental data of the whole-blood infection assays with *C. albicans* (see Materials and Methods section for details). The mean values of the transition rates could be estimated with standard deviations below 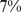, indicating the high accuracy of the fitting procedure (Table 1) and the comparison of simulated and experimental data clearly showed quantitative agreement for the whole time course of infection (Fig. 5). The simulations were repeated 100 times for the normally distributed transition rates (Table 1) and the thickness of the solid lines in Fig. 5 represents the mean 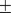 standard deviation due to these variations. The limiting value of the standard deviations was below 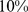 for each quantity and the solid lines remained well within the experimental error bars, indicating that the simulation results are robust against variations in the transition rates.

**Table 1:** Transition rates of the state based model.

	rate	standard deviation	standard deviation [%]
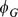	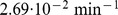	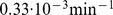	1.24
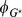	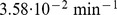	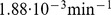	5.24
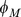	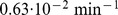	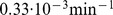	5.25
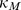	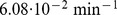	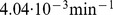	6.64
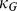	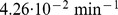	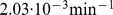	4.76
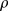	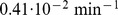	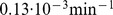	3.25
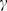	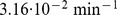	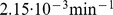	6.8
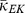	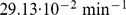	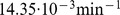	4.93

The transition rates of the state-based model are given by the phagocytosis rate 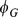 of PMN that phagocytose for their first time, the phagocytosis rate 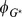 of PMN that phagocytose for at least the second time, the phagocytosis rate 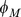 of monocytes, the intracellular killing rate 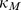 of monocytes, the intracellular killing rate 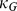 of PMN, the resistance rate 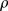 and the rates that determine the extracellular killing 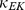 and 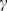.

### *C. albicans* is mainly eliminated from human blood via PMN phagocytosis

**Figure 6:**
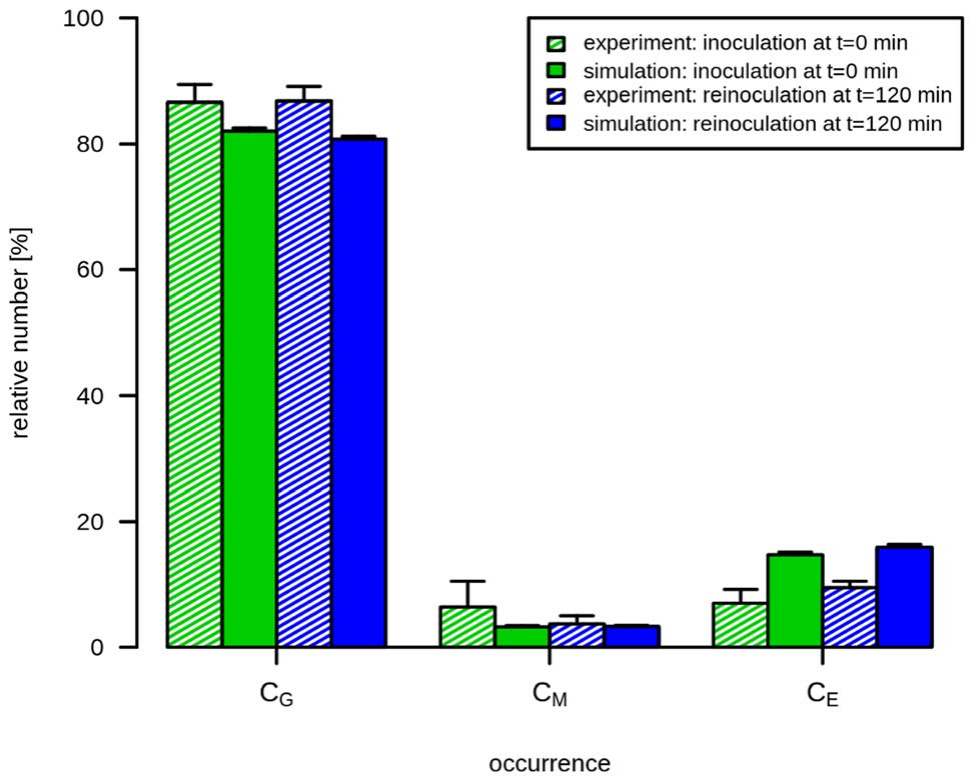
Simulation *versus* experimental results of reinoculation of alive *C. albicans* cells. Results of inoculation of *C. albicans* into human whole blood at 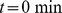 and 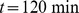 (blue bars). At both time points, 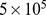 *C. albicans*/ml were inoculated in human whole blood and the FACS analysis was performed at 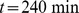. This analysis provides the relative number of *C. albicans* cells that were phagocytosed by PMN (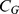) or by monocytes (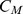) or those who remained in extracellular space (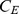). For the comparison with primary inoculation of *C. albicans*, 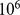 *C. albicans*/ml were inoculated and analyzed by FACS at 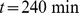 (green bars). The experimental conditions were also applied for the simulation with estimated parameters. Filled bars refer to the simulation results and striped bars indicate data obtained by FACS analysis.

**Figure 7:**
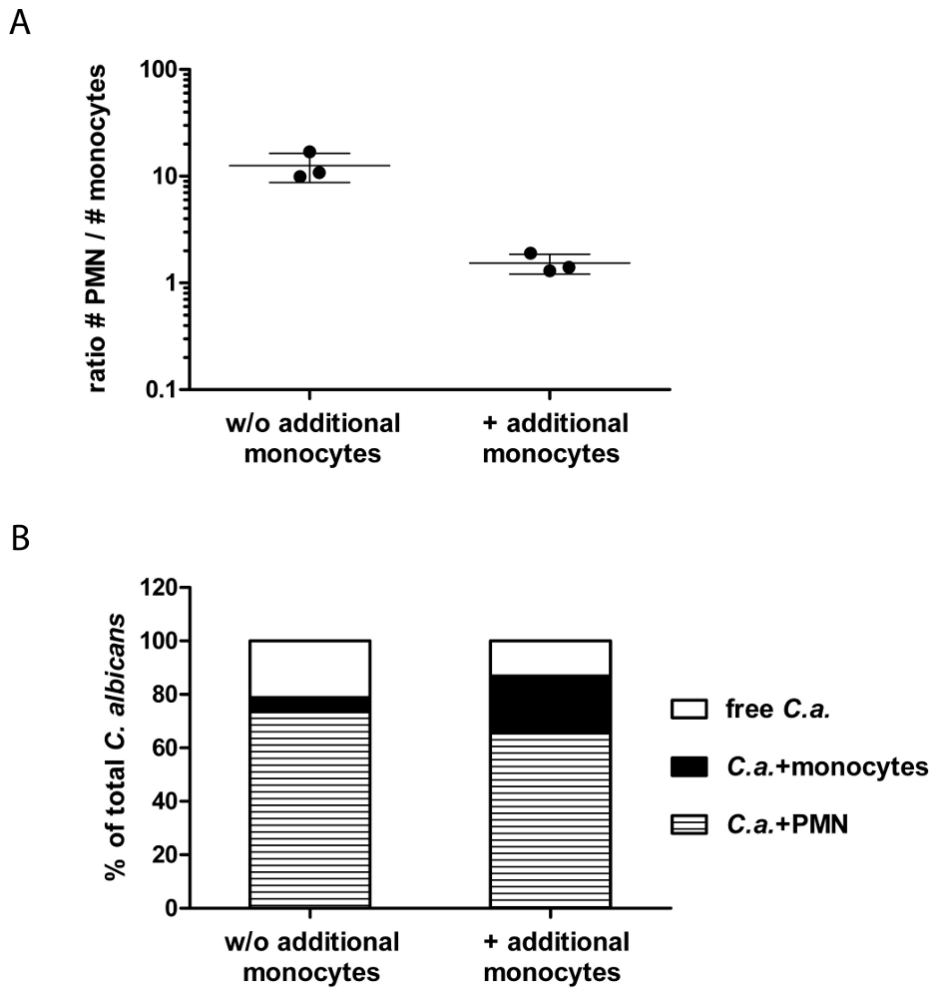
PMN are more potent in *C. albicans* phagocytosis than monocytes. To investigate the influence of a changed PMN to monocytes ratio on the interaction of *C. albicans* with innate immune cells human whole blood was infected with GFP-expressing fungal cells for 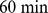 in the absence or presence of additional monocytes from the same donor. (A) Increased total monocyte amounts resulted in reduced PMN to monocytes ratios compared to whole-blood samples without (w/o) additional monocytes. Each dot represents the ratio of PMN to monocytes of independent experiments with blood from different donors. The mean 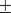 standard deviation is given for each experimental condition. (B) During whole-blood infection with increased monocyte amounts *C. albicans* still predominantly associates with PMN. The percentages of *C. albicans* associated with PMN (striped bars) or monocytes (black bars) were calculated relative to total *C. albicans* cells in blood (set to 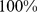). All values correspond to the means of three independent experiments with whole blood from different donors.

Due to experimental limitations it is impossible to quantify the contribution of single effector mechanisms to the overall elimination of *C. albicans* in the whole-blood infection model, whereas the virtual infection model allowed separate analyses for all killing mechanisms. In the state-based model, we assumed that the phagocytosis rates were constant in time. This assumption was experimentally justified by reinoculation of *C. albicans* cells into an infected blood sample after 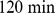. Since we observed a similar distribution pattern for the newly added *C. albicans* after 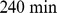 as in the initial experimental set-up (Fig. 6), it could be concluded that the phagocytosis rates remain fairly constant over time. According to the model, phagocytosis of *C. albicans* by a monocyte is less probable than uptake by PMN (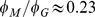). To confirm the different phagocytic capacity of PMN and monocytes we experimentally increased the total monocyte number by adding autologous isolated monocytes to blood samples. Distribution of *C. albicans* to the different immune cell populations in these samples was quantified after 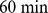 and compared to non-substituted blood samples. Despite an almost equal number of PMN and monocytes in the substituted blood samples (PMN to monocytes ratio: 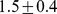), the majority of *C. albicans* cells still associated with PMN (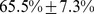), clearly indicating that PMN are more efficient in taking up *C. albicans* than monocytes (Fig. 7). In addition, the model predicted that internalization by PMN that phagocytose for the first time is lower compared to internalization by PMN which did phagocytose more than one *C. albicans* cell (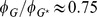). We examined the robustness of the prediction 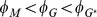 by performing four restricted parameter estimations with conditions (i) 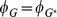, (ii) 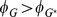, (iii) 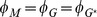 and (iv) 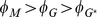. For all those conditions, the fitting errors were significantly larger than the fitting error of free parameter estimation (see Fig. S2A). This was verified by Wilcoxon rank-sum test and the variations in the corresponding parameter sets are depicted in Fig. S2B.

Surprisingly, the model predicted that intracellular killing of PMN occurs with a lower transition rate than intracellular killing by monocytes (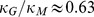). To test the robustness of this prediction we repeated the parameter estimation procedure under the biologically motivated condition 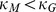. We found that the fitting error of this conditional parameter estimation was not significantly different from the free parameter estimation, but is again significantly smaller than that of parameter estimations under conditions (i)–(iv) (see Fig. S2A). The parameter estimation with condition 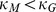 yielded 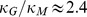, which was mainly due to a decrease of 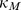 by more than 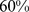. This was compensated by relatively small variations in all other rates (see Fig. S2B), indicating that the parameter estimation for the virtual infection model is generally robust in all the other rates.

The original parameter estimation revealed that most *C. albicans* cells were killed within PMN (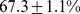), 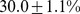 were killed extracellularly and a small amount was killed within monocytes (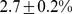). Consequently, elimination of *C. albicans* in human blood is mainly mediated by PMN which – apart from being present in higher numbers – release antimicrobial peptides inducing extracellular killing and are therefore more effective in eliminating *C. albicans* than monocytes.

### Dynamic distribution of *C. albicans* in immune cells is accurately predicted by virtual infection model

**Figure 8:**
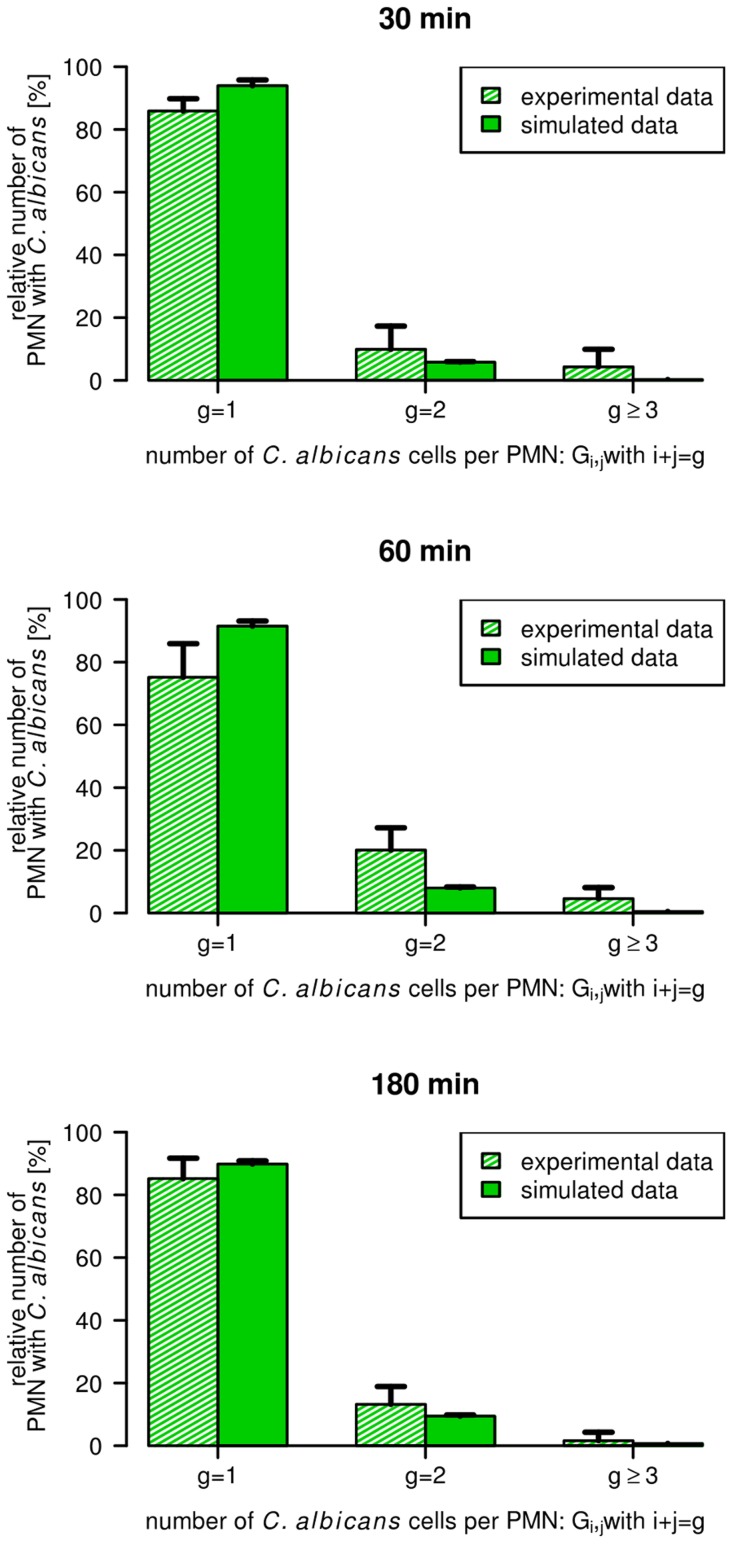
Number of *C. albicans* cells per PMN that contain *C. albicans*. Time course of the relative number of PMN that contain one (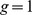), two (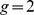) or at least three (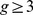) *C. albians* cells that are alive and/or dead, *i.e.* 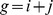, with 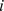 alive and 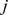 killed *C. albicans* cells. Striped bars refer to experimental results obtained by manually counting *C. albicans* in microscopic images and filled bars indicate corresponding simulation results.

The virtual model allowed us to distinguish between intracellularly and extracellularly killed *C. albicans* cells inside monocytes and PMN. Both immune cell types bear more intracellularly killed than extracellularly killed *C. albicans* throughout the first 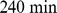 of infection (PMN 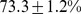 versus 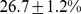, monocytes 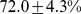 versus 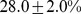, see Fig. 5). To analyze the average contribution of single PMN to elimination of *C. albicans* we determined the distribution of alive and killed *C. albicans* over PMN. The model predicted PMN to phagocytose up to five viable *C. albicans* cells, with most of the PMN containing one fungus (see Fig. S3A). The amount of PMN that contain viable *C. albicans* started to decrease after 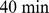, whereas the amount of PMN containing killed *C. albicans* increased and reached a maximum after 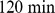 (see Fig. S3B). We found that PMN contained at maximum six *C. albicans* cells, however, the majority of cells carried only one. After 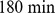, the relative amount of PMN that contained one *C. albicans* cell was predominantly greater than the fraction of PMN that contained more than one *C. albicans* cell (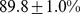 versus 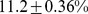, see Fig. S3C). Similar results were obtained for the distribution of *C. albicans* in monocytes (Fig. S4). These predictions were experimentally verified by manually counting *C. albicans* cells per PMN in blood smears with quantitatively comparable results, confirming that most PMN which had phagocytosed contained a single *C. albicans* cell throughout the experiment (see Fig. 8). Excellent fits were achieved for 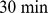 and 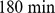 after inoculation whereas a higher degree of variation was observed at 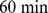 after inoculation, consistent with a higher standard deviation of the experimentally quantified concentrations around this time point (see Fig. 8). These data indicate that activation of PMN triggered by phagocytosis of *C. albicans* enhances extracellular killing and results into a series of secondary phagocytosis events. Therefore, the distributions of *C. albicans* cells in PMN and monocytes deviate from the distributions expected for simple Poisson statistics. A comparison revealed a decrease in the number of monocytes containing *Candida* cells, whereas the number of PMN containing two or more *Candida* cells was increased (see Supporting Information Text S1 and Fig. S5). These deviations are a direct result of the relationship 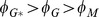.

### *C. albicans* escapes phagocytosis

Experimental results had shown that a fraction of *C. albicans* cells remained extracellular and some fungi also survived throughout the experiment (Fig. 5B). These findings could not be explained by proliferation of *C. albicans* as budding could not be observed and filamentation does not lead to an increase of cell numbers. Lytic escape from phagocytes, which has been described for *C. albicans* [31], could be excluded as no cell death occurred throughout the experiment. In the model, this was integrated by allowing extracellular *C. albicans* cells to become resistant against phagocytosis and further killing (Fig. S1). This was required for fitting the virtual infection model to the experimental data as the fractions of extracellular and viable *C. albicans* cells were not negligible.

**Figure 9:**
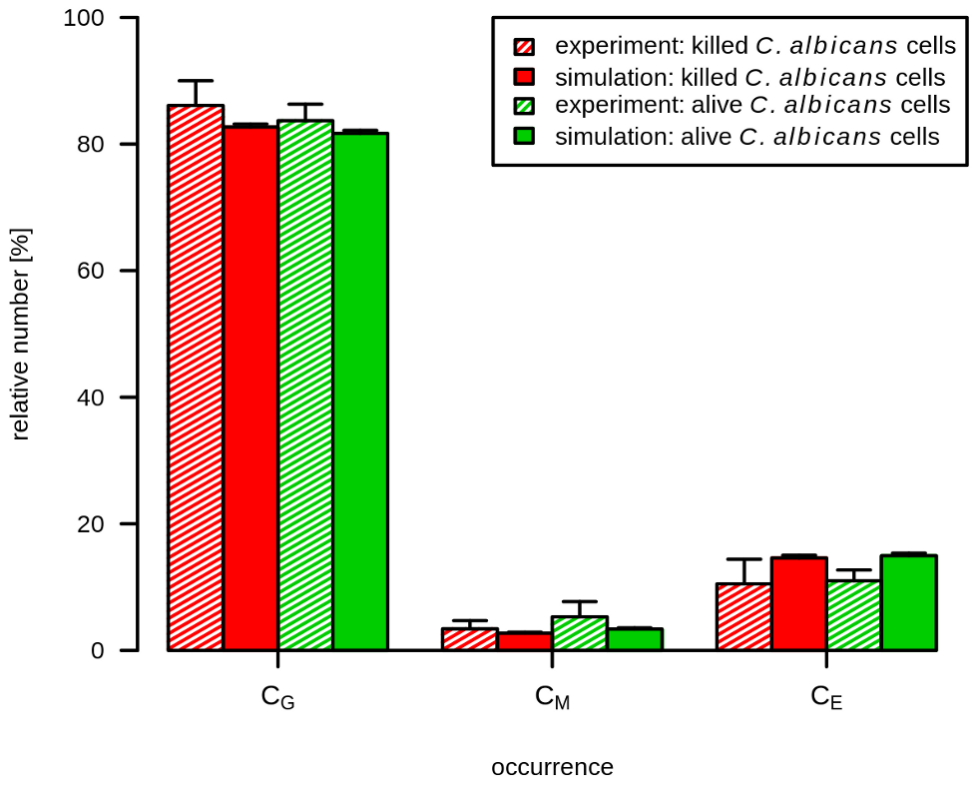
Simulated and experimental results of inoculation of killed *C. albicans* cells. Samples of human whole blood were infected with previously killed *C. albicans*. At 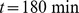 after inoculation, the relative number of killed *C. albicans* in PMN (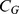), monocytes (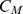) and extracellular space was measured by FACS analysis. Red bars indicate results of inoculation of 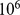 killed *C. albicans* per ml and green bars represent results of the initial experimental set-up, *i.e.* inoculation of 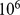 alive *C. albicans* per ml. Striped bars refer to experimental measured data with corresponding standard deviations. Filled bars show results of associated combinations of simulated data, that was generated with estimated transition rates and start conditions similar to the corresponding experimental set-up.

Our model predicted that almost all *C. albicans* cells that remained alive had developed resistance against phagocytosis and further killing (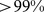) and only few fungi remained alive in PMN (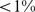) and monocytes (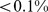). Resistant fungal cells also constituted the major fraction (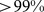) of extracellular *C. albicans* at 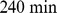 *post infection*. Using a non-filamentous mutant (*C. albicans efg1* 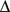, *cph1* 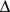) we could demonstrate that development of resistance was not linked to filamentation as this mutant showed an identical distribution as the wild-type without developing filamentous forms (distribution of *C. albicans* 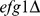, 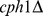 at 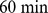 *p.i.* 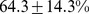 associated to PMN, 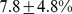 associated to monocytes and 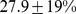 free, 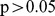 for all). Moreover, inoculation of killed *C. albicans* cells into human blood proved that killed fungal cells developed resistance against phagocytosis with identical rates as viable fungi resulting in similar amounts (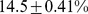 for viable versus 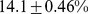 for inactivated *C. albicans*) of extracellular fungi (Fig. 9). The simulation results predicted that the amount of alive resistant *C. albicans* cells was larger than the relative number of killed resistant *C. albicans* cells, *i.e.* 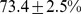 versus 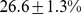, respectively, which was in line with the observation that extracellular *C. albicans* showed continued filamentous growth throughout the experiment. Development of resistance was not linked to exhaustion of the host cells. In contrast, immune cells in the model infection system clearly retained their phagocytic capacity throughout the experiment. This was shown by reinoculation of an infected blood sample after 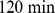, which resulted in identical uptake kinetics as primary infection (Fig. 6). To further confirm these data we added freshly drawn blood of the same donor to an infected blood sample to test whether the new immune cells were able to take up all or part of the extracellular resistant *C. albicans* population. As expected, no additional uptake of *C. albicans* cells could be observed. Taken together, the simulation results revealed that development of resistance against phagocytosis and further killing is the only way for *C. albicans* cells to survive immune activation in human blood.

## Discussion

We applied a state-based modelling approach to simulate the host-pathogen interaction for *C. albicans* in human blood. This approach allowed to set up a virtual infection model that captures the stochastic transitions between systems states, *e.g.* including all possible configurations of alive and killed *C. albicans* cells in monocytes and PMN as well as in the extracellular space. In contrast to deterministic models based on differential equations, the bookkeeping of discrete transitions in the state-based model enabled us to accurately model (i) the killing by secreted antimicrobial factors due to the primary phagocytosis of *C. albicans* cells by PMN and (ii) the dynamic distribution of killed and alive *C. albicans* in immune cells. This is a consequence of the fact that non-spatial agent-based models represent interactions between cells occurring in small numbers as stochastic events and allow for decision making depending on the preceding occurrence of specific events [27].

*A priori* unknown transition rates between any two states could be estimated by fitting the simulation results to the experimental data using the Monte Carlo method of simulated annealing. This procedure enabled us to quantify transition rates with high accuracy by identifying the set of parameters that globally minimizes the least-square error between the results of the simulation and the experiment. The current model has been fitted to results obtained with blood samples from several independent blood donors. Furthermore, we have shown that overall distribution rates are highly similar for a set of unrelated clinical bloodstream isolates. Despite this, it has to be noted that our data will most likely underestimate the biological variability of both host and pathogen as a small set of selected donors and *C. albicans* strains does not cover the complete biological variability of both populations. However, our approach offers an unique option to study this diversity, *e.g.* by using *C. albicans* strains that have been shown to differ in their interaction with host immunity [32]. In addition, the ability to use the whole-blood infection assay rather than purified primary immune cell populations bears several other advantages: (i) as no isolation procedure is involved all cells in the assay are completely untouched and should show minimal pre-activation [33], (ii) the whole-blood model allows communication between different effector cells and contains a functional complement system [12], [17], [34], (iii) the whole-blood model enables pharmacological intervention by blocking several arms of innate immune activation [35], [36]. Consequently, several future applications of our approach can be envisioned. These include the comparative analysis of different pathogens, investigation of clinically relevant scenarios (neutropenia) as well as studies on the influence of genetic markers on innate immune activation.

The virtual infection model clearly predicts a predominant role of neutrophils in the early immune response mounted in human blood against *C. albicans*. Although neutrophils have mostly been considered as central in the defense against invasive *C. albicans* infection, their role in the clinical setting is not unambiguous. In patients with chronic granulomatous disease, a congenital disorder of NADPH oxidase which prevents oxidative burst and formation of NETs, candidemia is surprisingly rare, especially when compared to invasive mould infections like aspergillosis or zygomycosis [37], [38]. In line with this, many studies have failed to identify neutropenia as an independent risk factor for candidemia [39]. As these studies have largely been performed in ICU settings, this may however be due to the rarity of neutropenic patients in these cohorts. In cancer patients, neutropenia has been found to contribute to the risk for developing candidemia [40]–[42] and it is generally accepted that the outcome of candidemia is impaired in neutropenic patients and therefore current therapeutic guidelines recommend intensified treatment protocols for candidemia in neutropenic patients [43].

Our results suggest that neutrophils are of central importance in the immediate response against invading *C. albicans* and contribute to elimination in two ways. First, they effectively take up viable *C. albicans* cells and kill them intracellularly. This activity of neutrophils has generally been considered a major route of antifungal activity and was studied in detail using purified neutrophils [10], [44]–[46]. Second, neutrophils release antifungal effector molecules upon activation that result in extracellular killing of *C. albicans*. Our model predicts that both mechanisms together account for as much as 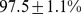 of fungal killing. This clearly underlines the outstanding importance of neutrophils in mounting a protective response against invasive *C. albicans* infection which has been suggested by experimental *in vivo* studies [47]. Bloodstream infection with *C. albicans* frequently results in organ dissemination, which can affect many organs and anatomical sites including liver, eye, joints and even brain. In an early study, 9 of 32 patients with candidemia showed chorioretinitis compatible with *Candida* infection and routine performance of fundoscopy is advised for patients suffering from candidemia within one week of treatment initiation [43], [48]. Other studies also documented high rates of dissemination in candidemia, resulting in a disease entitity termed acute disseminated candidiasis [49], [50]. Interestingly, profound and prolonged neutropenia can result in a different disease entity known as chronic disseminated candidemia which is defined by a hematogenous infection of liver and spleen by *Candida* spp. [51]. Our virtual infection model suggests that elimination of *C. albicans* will be severely hampered in neutropenic blood, which could explain increased levels of dissemination in the respective patients. The ability of *C. albicans* to disseminate is linked to its ability to interact with endothelial cells in a way that allows invasion of tissue [52], [53]. However, to establish disseminated infection in multiple organs, it is a prerequisite that some *C. albicans* cells remain viable in the blood for a prolonged time period. Here, we provide clear evidence that this is indeed the case. Furthermore, of several hypotheses that could potentially explain long-term survival of *C. albicans* in human blood, the model clearly predicts the development of resistance against phagocytosis among an extracellular population of fungal cells to be the most favourable explanation. The molecular basis for development of resistance will have to be addressed in future studies. However, experimental testing of model-generated hypotheses has provided some important clues: (i) development of resistance against phagocytosis does not require viability of the fungus. In contrast, thimerosal-killed yeast cells were able to acquire resistance at identical rates as viable fungi. This also clearly proves that (ii) development of resistance is not linked to filamentation of *C. albicans*. In line with this finding, a non-filamentous 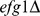, 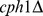 mutant of *C. albicans* was also able to acquire resistance at the same rate as *C. albicans* wild-type. (iii) Finally, the resistance phenotype does not seem to be linked to exhaustion of phagocytes at later stages of infection. This could be shown by reinoculation after two hours of initial infection, which again resulted in unimpaired phagocytosis and killing of the newly inoculated yeast cells.

A range of host factors has previously been shown to bind to the fungal cell wall and some *Candida* proteins may even recruit several host factors at a time [16], [54]. Shielding of the fungal cell wall by host factors may be the basis for developing resistance against phagocytosis and/or killing of *C. albicans* as observed in our model. Although so far no study has addressed the recruitment of host factors from complex and physiological environments, the established whole-blood infection model in combination with flow-cytometry assisted cell sorting offers a unique opportunity to pursue this hypothesis in future experiments. Moreover, interpreting the experimental results in the light of the virtual infection model will enable quantitative analyses of the dynamic immune response and the relative importance of defence mechanims by iterative cycles between experiment and theoretical modeling.

## Materials and Methods

### Experimental methods

#### Ethics statement

Human peripheral blood was collected from healthy volunteers after informed consent. This study was conducted according to the principles expressed in the Declaration of Helsinki. All protocols were approved by the Ethics Committee of the University Hospital Jena (permit number: 273-12/09) written informed consent was obtained from all blood donors.

#### Fungal strains and culture

*Candida albicans* wildtype (SC5314) was used for all experiments. For construction of CaGFP (*ADH1/adh1::GFP-SAT1*) we transformed a cassette including a *C. albicans* optimized *GFP* from the vector pNIM1 [55] and *SAT1* as selection marker [56] as well as homology regions for integration into the *CaADH1* locus into the *C. albicans* wild type strain SCR5314, using lithium acetate protocol [57]. Transformants were grown for two days on YPD with 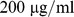 nourseothricine and verified by PCR and microscopy. For an infection of whole blood, *C. albicans* was grown over night in YPD-medium (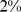 D-glucose, 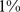 peptone, 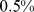 yeast extract in water) at 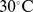, reseeded in YPD-medium, grown for five hours at 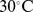 into the mid-log-phase, and harvested in HBSS. *C. albicans* yeasts were killed by incubation in 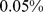 thimerosal (Sigma-Aldrich) in HBSS at 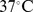 for 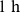 and then rinsed extensively.

#### Whole-blood model

For anticoagulation of blood samples we used 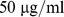 lepirudin (Refludane, Cergene), a recombinant hirudin that does not influence complement activation [58]. HBSS (for mock-infection control) or *C. albicans* in appropriate concentrations of yeast cells per ml whole blood were added and further incubated for various time points (as indicated) at 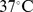. After incubation, samples were immediately subject to flow cytometry or other analyses. To collect plasma samples, whole-blood aliquots were immediately placed on ice, centrifuged (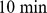, 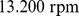, 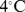) and plasma was stored at −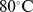 until further analysis.

#### Monocyte isolation

Human monocytes were isolated from peripheral blood of healthy volunteers. First, primary human peripheral blood mononuclear cells (PBMCs) were isolated by density gradient centrifugation using BIOCOLL (Biochrom AG). Monocytes were seperated from PBMCs by positive magnetic bead selection via magnetic cell sorting system (MACS) using human CD14 MicroBeads (Miltenyi Biotech) according to the manufacturer's instructions.

#### Flow cytometry

Analyses of immune cell populations in whole blood with regard to phagocytosis of fungal cells or their expression of cell surface activation markers were performed using differential FACS staining and subsequent measurement with a FACS Canto II. To distinguish different immune cells, 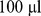 whole blood were stained with mouse anti-human CD3-PerCP (clone SK7, T cells), CD19-APC (clone HIB19, B cells), CD56-V450 (clone B159, NK-cells) and CD66b-V450 (clone G10F5, PMN) obtained from BD. Monocytes were labeled with mouse anti-human CD14-PerCP antibody (clone 47-3D6, Abcam). Changes in surface expression were investigated for the early activation antigen CD69 (mouse anti-human CD69-PE, clone F50), 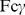 receptor I (mouse anti-human CD64-APC, clone 10.1), 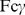 receptor III (mouse anti-human CD16-APC, clone 3G8) and CD11b subunit of CR3 (mouse anti-human CD11b-APC, clone ICRF44) using antibodies obtained from BioLegend. The stained samples were treated with BD FACS Lysing solution that lyses erythrocytes while preserving and fixing leukocytes, followed by washing and harvesting cells in BD CellWASH solution.

FlowJo 7.6.4 software was used for analysis. The strategy used to evaluate the association of *C. albicans* to immune cells in human blood is shown in Fig. S6.

#### Oxidative burst

The PMN oxidative burst was measured using commercially available Bursttest (Orpegen Pharma). Immediately after incubation, 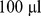 whole blood were treated according to the kit procedures. Results were expressed as median fluorescence intensity of the whole PMN population.

#### Quantification of secreted proteins

The concentrations of cytokines (Bio-Plex Pro Human Cytokine 27-plex Assay, Bio-Rad) and antimicrobial peptides (MILLIPLEX MAP Human Sepsis Magnetic Bead Panel 3, Millipore and Procarta Immunoassay Human Myeloperoxidase, Affymetrix) within plasma samples were determined using Luminex technology. The analyses were performed according to the instructions from the manufacturer.

#### Preparation of Giemsa-stained blood smears

Blood smears were obtained from *C. albicans*-infected blood samples after various time points (as indicated). Smears were fixed and stained with 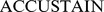 Giemsa stain (Sigma-Aldrich).

#### Statistical analyses

For all experiments, at least 4 independent replicates using cells from non-identical donors were used. Data are presented as arithmetic means 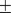 standard deviation and statistical significance (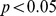) was calculated using a two-sided t-test for unpaired samples.

### Mathematical modeling

#### State-based model for the immune response against *C. albicans* in human blood

The state-based model comprises states symbolized by 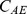, 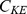, 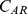 and 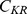 that refer to extracellular *C. albicans* cells being alive, killed and resistant, respectively (Fig. 3). Alive and killed *C. albicans* cells may become resistant or will be phagocytosed by monocytes 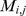 and granulocytes 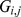 and may possibly become killed intracellularly. The two indices refer to the numbers 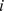 and 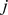 of internalized *C. albicans* cells that are alive and killed, respectively, and allow for the proper bookkeeping of intracellular processes. We checked that setting the range of these indices to 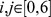 provided sufficient capacity for unrestricted phagocytosis, *i.e.* the distribution was not affected by these boundary conditions.

Phagocytosis by monocytes can be effective for both alive and extracellularly killed *C. albicans* cells with the same transition rate 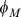. With regard to phagocytosis by PMN the transition rate is assumed to depend on whether a specific PMN did phagocytose once before or not [30]. In the case of extracellular killing by antimicrobial factors the transition rate 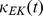 is proportional to the product of the constant transition rate 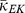 and the number of first-time phagocytosis events 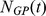 per PMN granulocytes 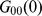:
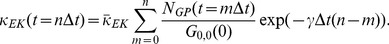
(1)Here, 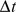 denotes the time step of the simulation and the time-dependent effect of antimicrobial factors, which is mediated by the monotonically increasing number 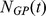, is associated with a half-life time that is characterized by the rate 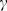.

The flow-diagram of the simulation algorithm is presented in Fig. S1 and was organized in a randomized fashion while ensuring that each *C. albicans* cell and each immune cell is updated only once per time step. In each time step we randomly choose the order in which immune cells are updated with regard to intracellular killing (step 1) or extracellularly killed *C. albicans* cells are phagocytosed or become resistant (step 2). Afterwards, alive extracellular *C. albicans* cells are updated with regard to one of the four possibilities (step 3): (i) phagocytosis by immune cells, (ii) transition to resistance, (iii) extracellular killing by antimicrobial factors, or (iv) continuance in the current state. The three steps are depicted in the top left box of Fig. S1. Note that performing step 1 and 2 in random order, followed by step 3, is crucial to avoid multiple updating of a state during one time step.

Each of the three steps involves a random decision making, *e.g.* with regard to the execution of a state transition and the choice of an immune cell to interact with. The random choice of an immune cell from an occupied state is depicted in the bottom right box of Fig. S1. First, the relative amount of monocytes versus PMN serves as a threshold to randomly decide about the immune cell type. Second, the distribution of all individuals of this immune cell type is sampled by a Monte Carlo acceptance-rejection method [59] to ensure that repeated random choices represent the immune cell distribution. The execution of a transition between two states 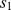 and 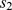 is realized by randomly choosing a real number 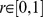 that is compared with the corresponding transition probability. The latter is related to the transition rate 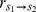 and the time step 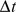 of the simulation as follows:
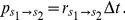
(2)This means that the transition rate is defined as the probability to change from state 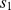 to state 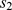 within the time step 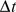. Note that the inverse of the transition rate defines the average time the transition from state 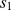 to 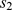 takes place, with the assumption that no other transition is available. In case 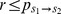, the transition will be performed, *i.e.* the number of individuals of these states will be reallocated with respect to the executed transition type (see boxes 1, 2 and 3 in Fig. S1).

Simulations were performed for a time-course of four hours, 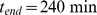, and with a time step of 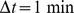. Initially, immune cells occupied states 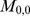 and 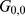, while 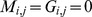 for all combinations of indices that are different from 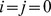. The initial number of individuals of immune cell states are determined according to average physiological numbers in blood.

#### Parameter estimation by the method of simulated annealing

We applied the method of simulated annealing based on the Metropolis Monte Carlo scheme [59]–[61] to estimate the unknown transition rates of the state-based model. This method randomly explores the parameter space of transition rates to find the global minimum of the fitting error, *i.e.* the most suitable parameter set that produces the best fit of the simulations to the data obtained from the whole-blood infection assay.

The parameter estimation algorithm starts with a randomly chosen parameter set 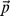. Next, the time-evolution of the state-based model was computed by the simulation algorithm using these parameters and the kinetics of various states was combined for comparison with experiment. These five quantities are referred to as *combined units* 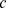 and are given by the extracellular *C. albicans* cells:
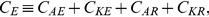
(3)phagocytosed *C. albicans* cells by monocytes:
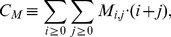
(4)phagocytosed *C. albicans* cells by PMN:
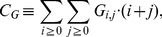
(5)killed *C. albicans* cells:
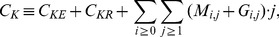
(6)and alive *C. albicans* cells:
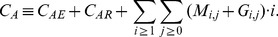
(7)Note that only three of the five combined units are independent of each other, because of the two conservation relations 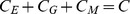 and 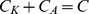. The combined units 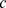 are obtained from the simulations and were scored by the least-squares error relative to corresponding experimental data points:
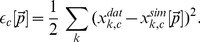
(8)Here, 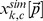 denotes the 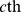 combined unit at the 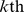 time point (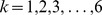) and 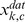 denotes the experimental data value as obtained from the whole-blood infection assay at this time point. For the scoring of the simulation result with parameter set 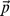, *i.e.* fitting of the model's five combined units, we calculated the fitting error as the weighted sum over the least-square errors
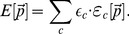
(9)Here, 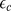 represents the weight of combined unit 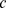 that was freely adjusted to achieve comparable accuracy of all combined units relative to their experimental data in a simultaneous fashion.

Next, the parameter set 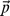 was randomly varied within a pre-defined neighborhood of 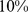, leading to a new set of parameter values, 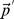. Subsequently, the simulation of the state-based model was run for 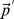 and the corresponding score 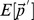 was calculated. Whether the new simulated data will be accepted or rejected is decided by applying the Metropolis Monte Carlo scheme. In case of a better fit by the parameter set 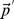, *i.e.* 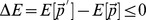, the new parameter set will be accepted, *i.e.* 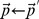 and the whole fitting procedure will be repeated. If the parameter set leads to a worse fit, *i.e.* 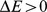, the Metropolis step will be performed, where the worse parameter set 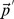 is only accepted if
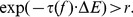
(10)Here, 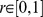 is a randomly chosen real number and 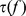 represents the inverse “system temperature” of the simulated annealing process. The simulation of the annealing process involves a gradual decrease of the system temperature with progressed fitting. This corresponds to an increase of 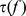 with the number of fitting steps 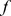 and, was implemented by a Hill function:
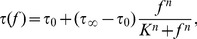
(11)where the Hill coefficient 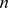 and the parameter 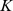 determine a smooth course of 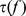 from 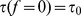 to 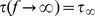.

On the one hand, acceptance of a worse parameter set prohibits being trapped in local minima of the fitting error. On the other hand, escape from a minimum becomes less probable with increasing fitting steps due to decreasing acceptance probability in the Metropolis step, *i.e.* the associated decrease in the simulated annealing of the system forces it into its equilibrium.

After performing the number of fitting steps, the fitting algorithm was repeated, *i.e.* it was started again after choosing a new random parameter set. This was done for a certain number of runs and the set of parameters with the minimal fitting error (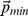) was saved from each fitting process. The mean values of the parameters and their standard deviations were computed over all runs to determine the robustness of the estimated parameters.

Due to the large numbers of immune cells and *C. albicans* cells in the whole-blood samples (see Table S1 in Supporting Information), the fitting procedure was organized in a stepwise fashion to decrease the computation time. We gradually increased the initial number of individuals 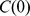, 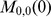 and 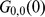 starting from a small system and increasing the system size step by step while keeping the ratio of these numbers constant. Parameter estimation was first performed for a small system with 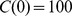, 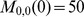 and 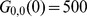 starting from a random parameter set. The resulting fitted parameter set was subsequently used as a starting point for a tenfold larger system until the experimental system with 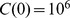, 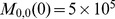 and 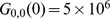 was reached (see Table S1 in Supporting Information). The fitting procedures were repeated at least 50 times for each system size.

## Supporting Information

Figure S1
**Flow-diagram of the simulation algorithm for the state-based model.** Course of simulated time-evolution of the state based model. At each time step 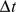, all individuals in *C. albicans* and immune cell (IC) state were considered for state transitions by comparison of the corresponding transition rate with a randomly chosen real number 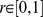. Box on the top left: The route of updating individuals of *C. albicans* (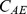, 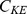, 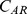 and 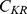) and IC states per time step 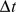, where steps 1) and 2) were performed in random order, followed by step 3). Box 1: Procedure of updating individuals of IC states in view of intracellular killing of *C. alicans*. The variable 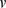 represents accepted transitions per individual of 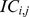 with 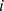 alive and 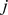 killed *C. albicans* that depend on the transition rate for intracellular killing (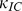). Box 2: Procedure of updating individuals of killed extracellular *C. albicans* (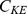) in view of phagocytosis by a randomly selected IC, depending on the rate of phagocytosis (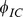), as well as in view of becoming resistant *C. albicans* (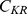) relative to the resistance rate 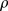. Box 3: Procedure of updating individuals of alive extracellular *C. albicans* (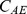). Here, all individuals of 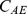 were tested for phagocytosis by a randomly selected IC, extracellular killing by antimicrobial factors as well as becoming resistant against phagocytosis, depending on the phagocytosis rate (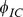), the extracellular killing rate (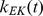) and the resistance rate (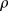), respectively. The three boxes on the bottom left depict the way of doing the test for the transitions phagocytosis, resistance and extracellular killing, where performing a transition depends on the comparison of the transition rate and a random real number 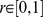. Box on the bottom right: Scheme of random selection of an immune cell state, were the relative frequency of both IC types (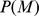, 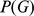) and the distribution of all IC state individuals (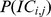) is taken into account.(TIF)

Figure S2
**Comparison of fitting errors and transition rates obtained by free and conditional parameter estimations.** (A) Fitting errors obtained from the parameter estimations under different conditions. The fitting error of the free parameter estimation (black bar) and of the parameter estimation with condition 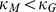 (red bar) are not significantly different (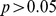, Wilcoxon rank-sum test). Parameter estimations with conditions 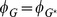 (blue bar), condition 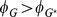 (pink bar), condition 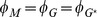 (green bar) and condition 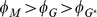 (orange bar) show significantly larger fitting errors with regard to both the free parameter estimation and the parameter estimation with condition 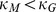 (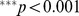, Wilcoxon rank-sum test). The error bars correspond to the standard deviations as obtained from repeated fitting procedures. (B) Transition rates determined from the free parameter estimation (black points) in comparison with transition rates from the parameter estimations with conditions 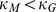 (red points), 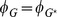 (blue points),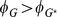 (pink points), 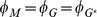 (green points) and 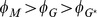 (orange points). Lines between the points do not represent values but are a guide for the eye. The solid lines refer to the free parameter estimation and the parameter estimation with condition 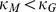 that do not have significantly different fitting errors. The dotted lines were used for all other parameter sets with significantly larger fitting error than the former two. All transition rate values are plotted in logarithmic scale. The error bars correspond to the standard deviations as obtained from repeated fitting procedures.(TIF)

Figure S3
**Time-dependent distribution of** ***C. albicans*** **cells in PMN.** The relative number of PMN containing at least one *C. albicans* cell over time is shown for their respective number of internalized *C. albicans* cells. (A) Time-dependent course of PMN that bear alive and killed *C. albicans* cells with respect to the number of alive *C. albicans* cells (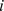). Here, 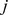 ranges from zero to six for PMN that contain 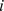 living *C. albicans* cells. (B) Time course of PMN that contain only killed *C. albicans* cells concerning the number of killed *C. albicans* cells in PMN. (C) Time course of PMN with at least one *C. albicans* cell regarding their total number of phagocytosed *C. albicans* cells (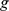), that is the sum of alive (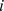) and killed (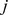) *C. albicans* cells.(TIF)

Figure S4
**Distribution of** ***C. albicans*** **in monocytes over time.** Relative number of monocytes that contain *C. albicans* over simulation time with respect to the number of internalized *C. albicans* cells. (A) Time dependent course of monocytes containing 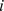 alive *C. albicans* cells with respect to the number of alive *C. albicans* cells. Here, the number of dead *C. albicans* (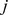) ranges from zero to the observed maximal number of killed *C. albicans* cells for monocytes containing 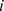 alive *C. albicans* cells. (B) Distribution of dead *C. albicans* cells in monocytes concerning the number of killed *C. albicans* cells per monocyte over time. (C) Time course of monocytes bearing *C. albicans* regarding the total number of *C. albicans* cells (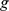). Here, 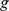 is defined as the sum of alive (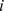) and killed (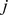) *C. albicans* cells.(TIF)

Figure S5
**Comparison of Poisson statistics and SBM simulation results for** ***C. albicans*** **distribution in immune cells.** Relative differences of Poisson statistics and SBM simulation results for different numbers of *C. albicans* cells per PMN (blue bars) and monocyte (yellow bars). The differences for the numbers of 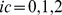 and 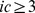 *C. albicans* cells per immune cell are shown. Free parameter estimation results are compared with simple Poisson statistics via its relative differences for different numbers of *C. albicans* cells per immune cell.(TIF)

Figure S6
**Flow cytometry gating strategy to investigate the distribution of** ***C. albicans*** **in human blood.** Representative flow cytometry plots illustrate the association of the fungus to immune cells 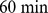 after inoculation. Total *C. albicans* cells were separated by the expression of GFP. (A) Within the gated *Candida*-GFP population we determined the association with monocytes (Mo, 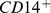) and PMN (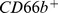). (B) We could not find any interaction of fungal cells with T-cells (TC, 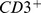), B-cells (BC, 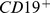) as well as NK-cells (NKC, 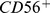).(TIF)

Supporting Information S1 Distribution of *C. albicans* cells in immune cells as expected from simple Poisson statistics.
(PDF)

Table S1
**Individual start conditions of the fitting algorithm.** Start conditions for the parameter fitting algorithm. The number of individuals of alive *C. albicans* cells in extracellular space (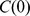), monocytes (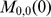) and PMN (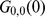) at time 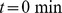 was stepwise increased by keeping their ratio constant. For each step, the number of runs with corresponding number of fitting steps per run and the range of 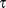 was adjusted.(PDF)
