# Supplementary figures and images for: A Virtual Infection Model Quantifies Innate Effector Mechanisms and Candida albicans Immune Escape in Human Blood
Source: PLoS Comput Biol. 2014 Feb 20;10(2):e1003479. doi: 10.1371/journal.pcbi.1003479 (PMC3930496; doi:10.1371/journal.pcbi.1003479)

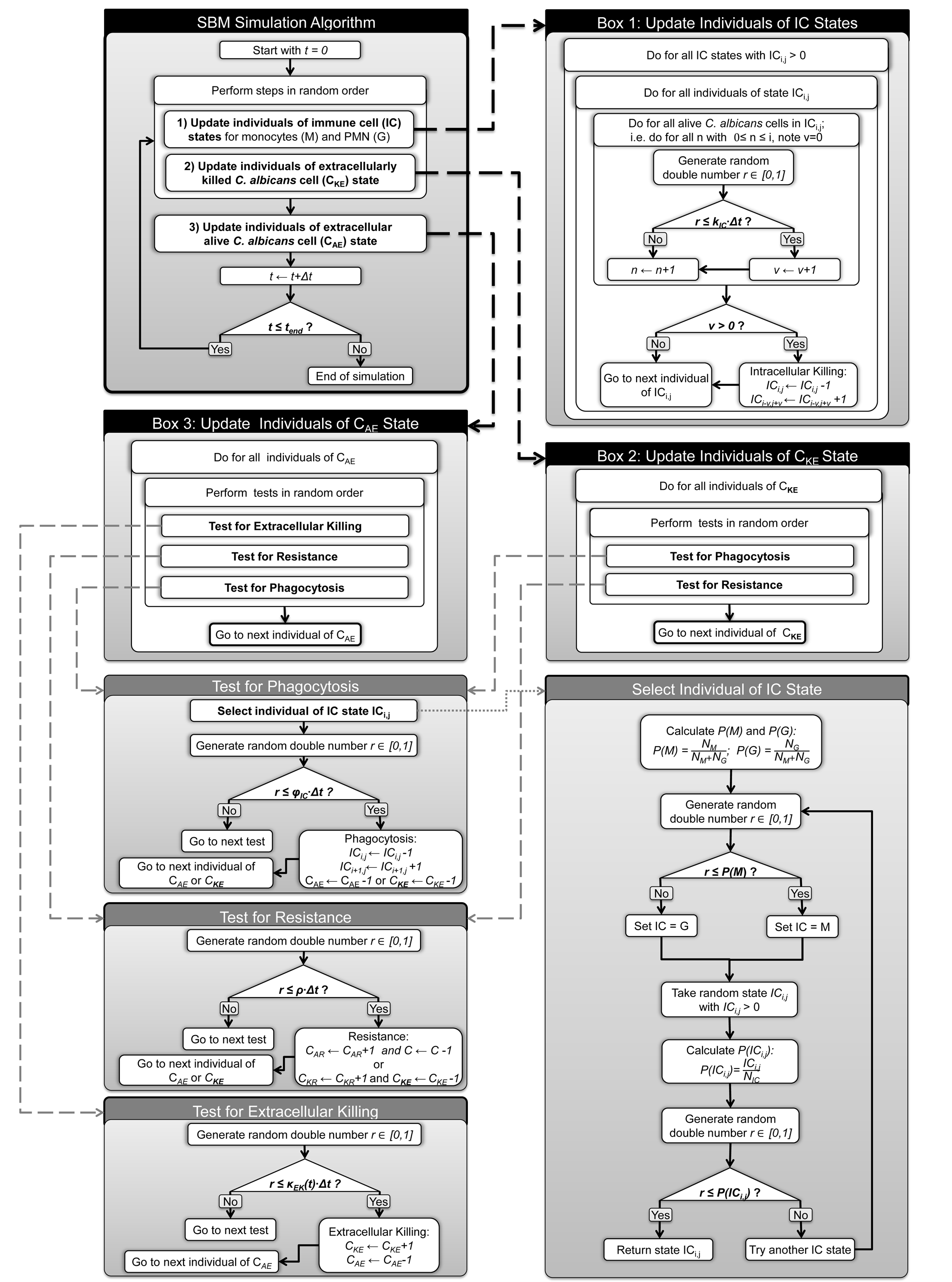

Supplement: Figure S1 — Flow-diagram of the simulation algorithm for the state-based model. Course of simulated time-evolution of the state based model. At each time step , all individuals in C. albicans and immune cell (IC) state were considered for state transitions by comparison of the corresponding transition rate with a randomly chosen real number . Box on the top left: The route of updating individuals of C. albicans (, , and ) and IC states per time step , where steps 1) and 2) were performed in random order, followed by step 3). Box 1: Procedure of updating individuals of IC states in view of intracellular killing of C. alicans. The variable represents accepted transitions per individual of with alive and killed C. albicans that depend on the transition rate for intracellular killing (). Box 2: Procedure of updating individuals of killed extracellular C. albicans () in view of phagocytosis by a randomly selected IC, depending on the rate of phagocytosis (), as well as in view of becoming resistant C. albicans () relative to the resistance rate . Box 3: Procedure of updating individuals of alive extracellular C. albicans (). Here, all individuals of were tested for phagocytosis by a randomly selected IC, extracellular killing by antimicrobial factors as well as becoming resistant against phagocytosis, depending on the phagocytosis rate (), the extracellular killing rate () and the resistance rate (), respectively. The three boxes on the bottom left depict the way of doing the test for the transitions phagocytosis, resistance and extracellular killing, where performing a transition depends on the comparison of the transition rate and a random real number . Box on the bottom right: Scheme of random selection of an immune cell state, were the relative frequency of both IC types (, ) and the distribution of all IC state individuals () is taken into account. (TIF) [file pcbi.1003479.s001.tif]

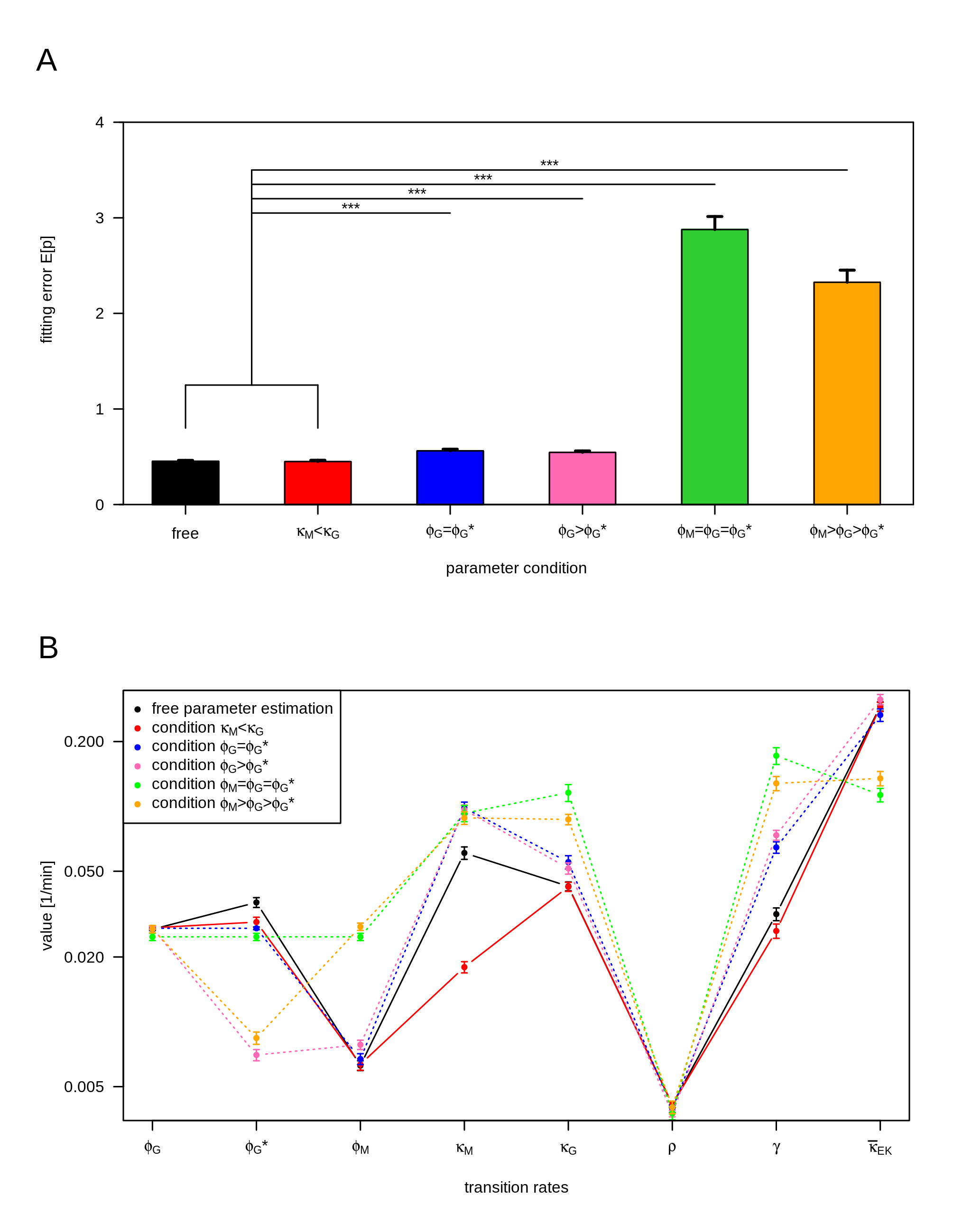

Supplement: Figure S2 — Comparison of fitting errors and transition rates obtained by free and conditional parameter estimations. (A) Fitting errors obtained from the parameter estimations under different conditions. The fitting error of the free parameter estimation (black bar) and of the parameter estimation with condition (red bar) are not significantly different (, Wilcoxon rank-sum test). Parameter estimations with conditions (blue bar), condition (pink bar), condition (green bar) and condition (orange bar) show significantly larger fitting errors with regard to both the free parameter estimation and the parameter estimation with condition (, Wilcoxon rank-sum test). The error bars correspond to the standard deviations as obtained from repeated fitting procedures. (B) Transition rates determined from the free parameter estimation (black points) in comparison with transition rates from the parameter estimations with conditions (red points), (blue points), (pink points), (green points) and (orange points). Lines between the points do not represent values but are a guide for the eye. The solid lines refer to the free parameter estimation and the parameter estimation with condition that do not have significantly different fitting errors. The dotted lines were used for all other parameter sets with significantly larger fitting error than the former two. All transition rate values are plotted in logarithmic scale. The error bars correspond to the standard deviations as obtained from repeated fitting procedures. (TIF) [file pcbi.1003479.s002.tif]

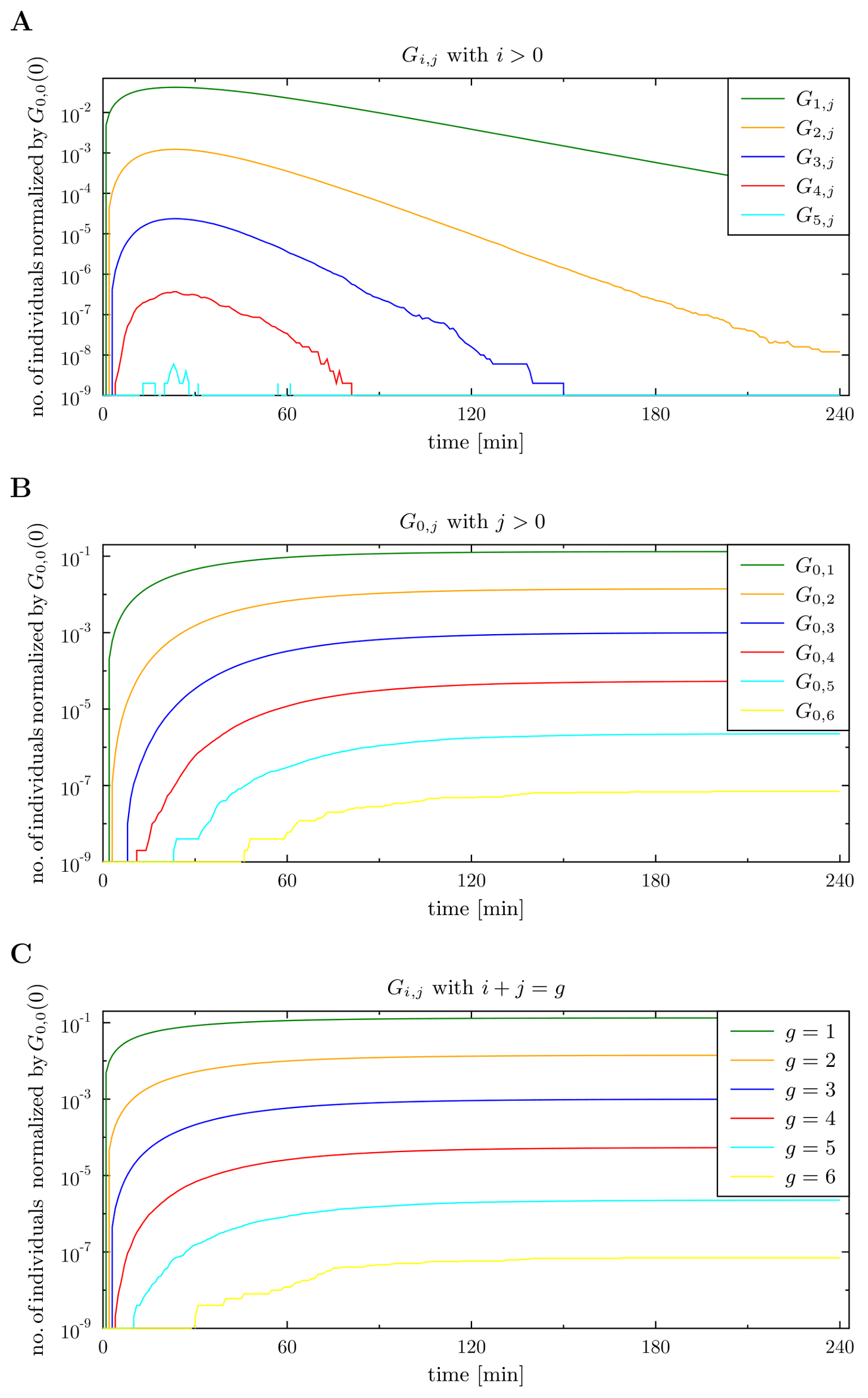

Supplement: Figure S3 — Time-dependent distribution of C. albicans cells in PMN. The relative number of PMN containing at least one C. albicans cell over time is shown for their respective number of internalized C. albicans cells. (A) Time-dependent course of PMN that bear alive and killed C. albicans cells with respect to the number of alive C. albicans cells (). Here, ranges from zero to six for PMN that contain living C. albicans cells. (B) Time course of PMN that contain only killed C. albicans cells concerning the number of killed C. albicans cells in PMN. (C) Time course of PMN with at least one C. albicans cell regarding their total number of phagocytosed C. albicans cells (), that is the sum of alive () and killed () C. albicans cells. (TIF) [file pcbi.1003479.s003.tif]

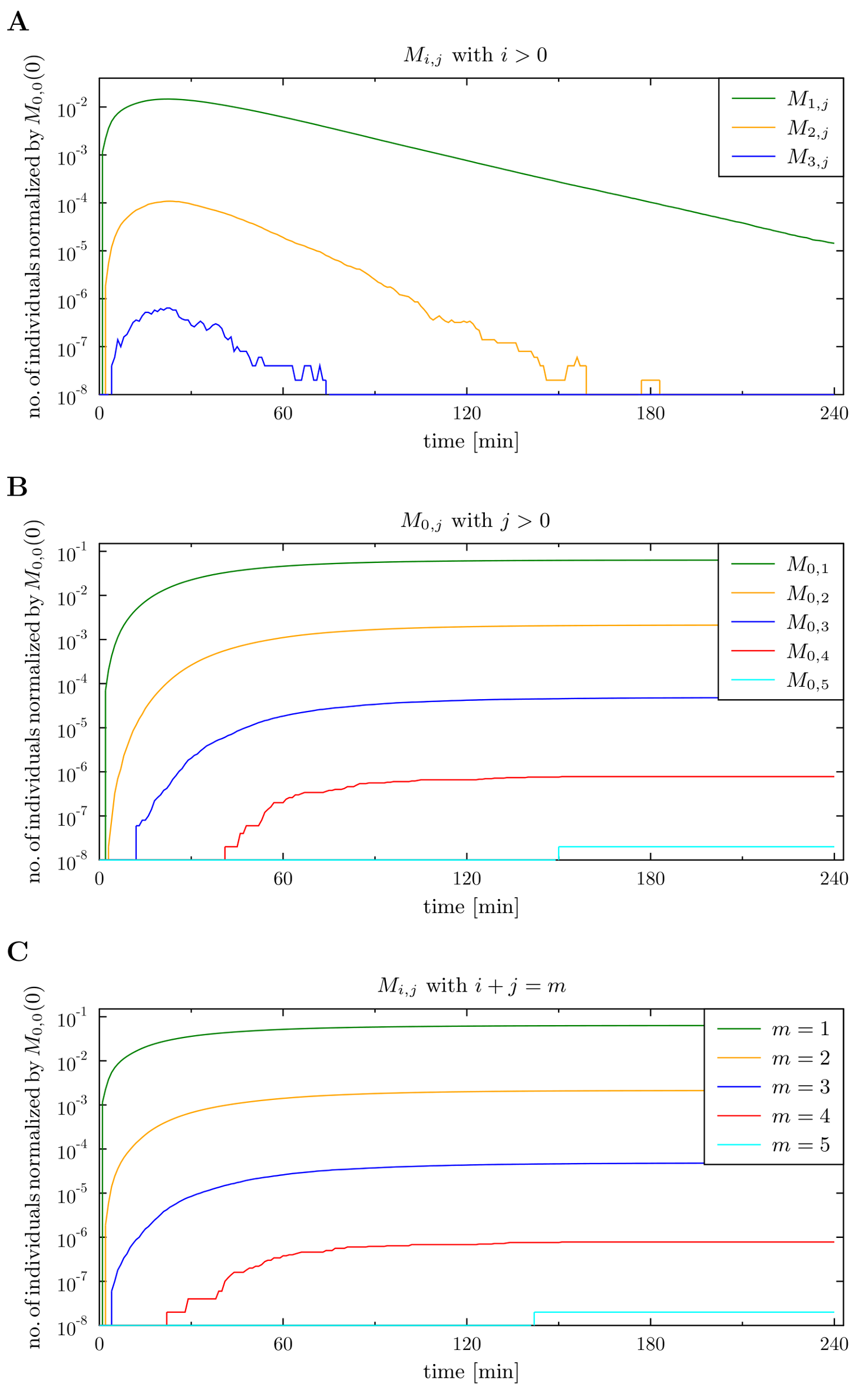

Supplement: Figure S4 — Distribution of C. albicans in monocytes over time. Relative number of monocytes that contain C. albicans over simulation time with respect to the number of internalized C. albicans cells. (A) Time dependent course of monocytes containing alive C. albicans cells with respect to the number of alive C. albicans cells. Here, the number of dead C. albicans () ranges from zero to the observed maximal number of killed C. albicans cells for monocytes containing alive C. albicans cells. (B) Distribution of dead C. albicans cells in monocytes concerning the number of killed C. albicans cells per monocyte over time. (C) Time course of monocytes bearing C. albicans regarding the total number of C. albicans cells (). Here, is defined as the sum of alive () and killed () C. albicans cells. (TIF) [file pcbi.1003479.s004.tif]

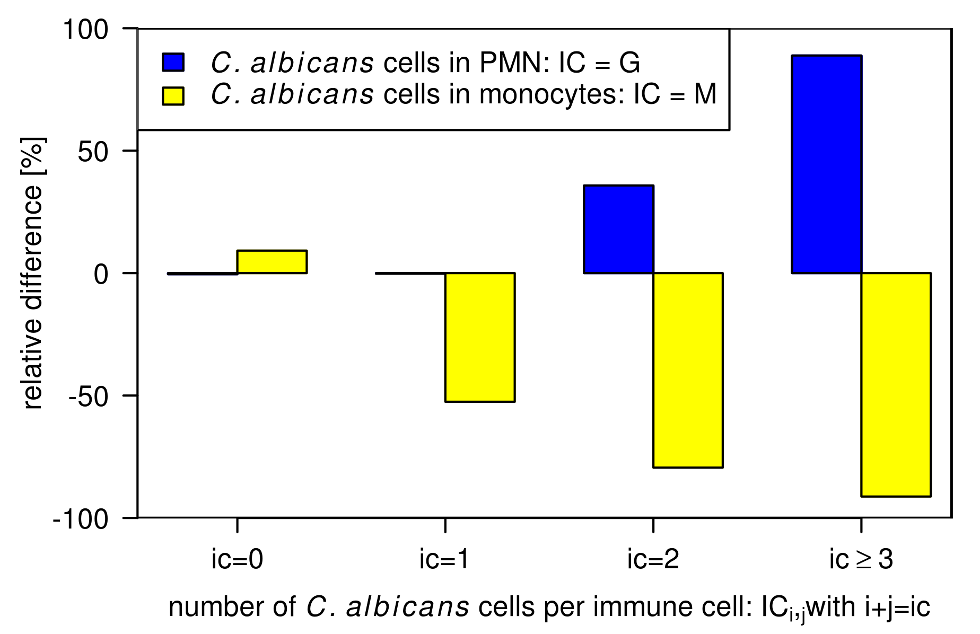

Supplement: Figure S5 — Comparison of Poisson statistics and SBM simulation results for C. albicans distribution in immune cells. Relative differences of Poisson statistics and SBM simulation results for different numbers of C. albicans cells per PMN (blue bars) and monocyte (yellow bars). The differences for the numbers of and C. albicans cells per immune cell are shown. Free parameter estimation results are compared with simple Poisson statistics via its relative differences for different numbers of C. albicans cells per immune cell. (TIF) [file pcbi.1003479.s005.tif]

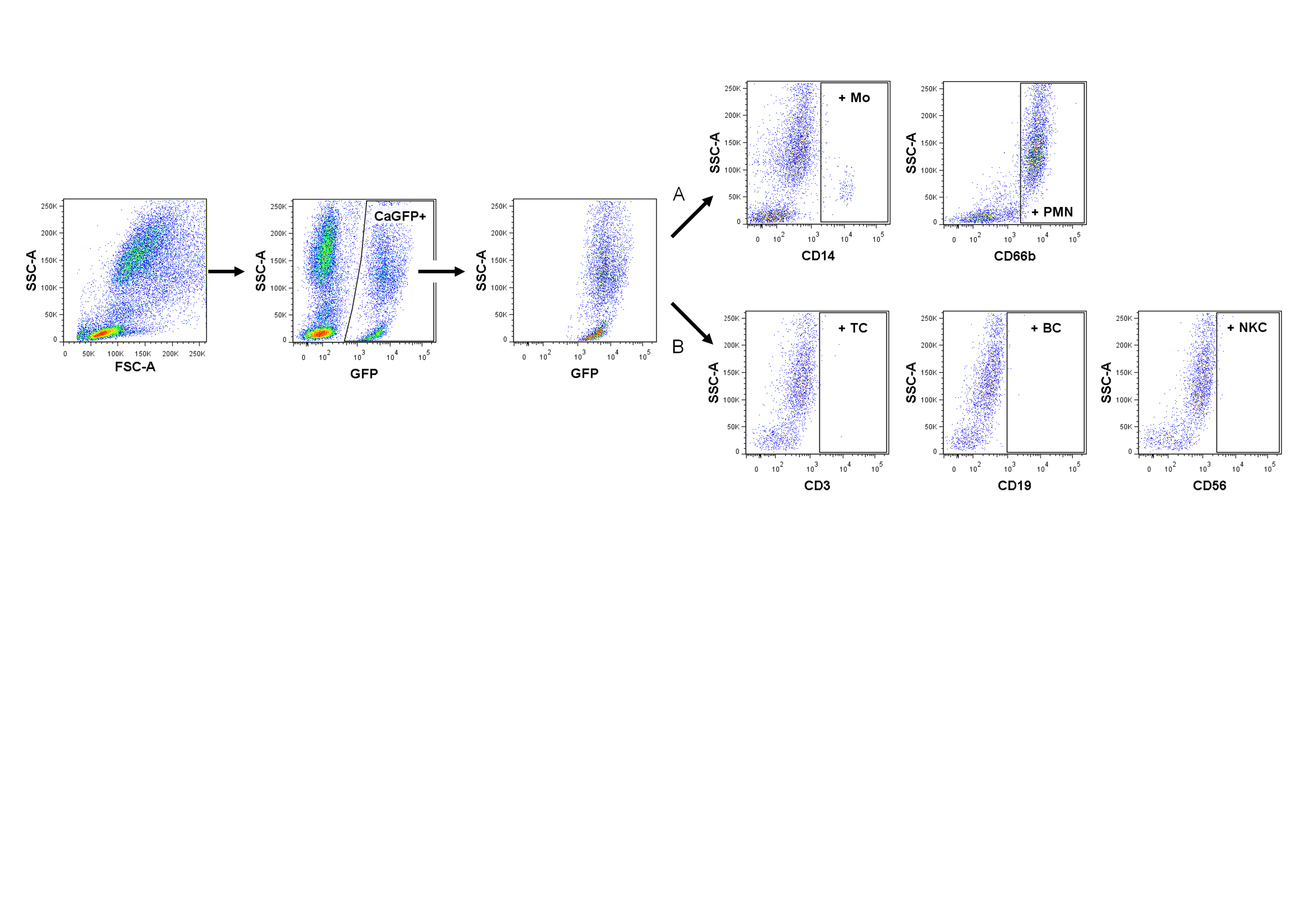

Supplement: Figure S6 — Flow cytometry gating strategy to investigate the distribution of C. albicans in human blood. Representative flow cytometry plots illustrate the association of the fungus to immune cells after inoculation. Total C. albicans cells were separated by the expression of GFP. (A) Within the gated Candida-GFP population we determined the association with monocytes (Mo, ) and PMN (). (B) We could not find any interaction of fungal cells with T-cells (TC, ), B-cells (BC, ) as well as NK-cells (NKC, ). (TIF) [file pcbi.1003479.s006.tif]
