# Supplementary material for: A Virtual Infection Model Quantifies Innate Effector Mechanisms and Candida albicans Immune Escape in Human Blood
Source: PLoS Comput Biol. 2014 Feb 20;10(2):e1003479. doi: 10.1371/journal.pcbi.1003479 (PMC3930496; doi:10.1371/journal.pcbi.1003479)
Supplement: Supporting Information S1 — Distribution of C. albicans cells in immune cells as expected from simple Poisson statistics. (PDF) [file pcbi.1003479.s007.pdf]

Supporting Information

Supporting Information S1: Distribution of *C. albicans* cells in immune cells as expected from simple Poisson statistics.

The equilibrium distribution of *C. albicans* cells in immune cells can be modelled as a series of Bernoulli experiments, where the total amount of *C. albicans* cells (C) comprises *C. albicans* cells in PMN (C_G), in monocytes (C_M) and cells in extracellular space that are resistant against phagocytosis and/or killing (C_R), *i.e.*

$$C = C_G + C_M + C_R\,. \tag{12}$$

The number of resistant *C. albicans* cells is defined as $C_R = f_R C$, where the fraction of resistant *C. albicans* cells (f_R) is obtained from the simulation of the state-based model (SBM). Similarly, the number of *C. albicans* cells in PMN and monocytes depends on the corresponding relative fractions, *i.e.* $C_G = f_G C$ and $C_M = f_M C$ with the condition $f_G + f_M + f_R = 1$. It follows that the relative fractions of *C. albicans* in PMN and monocytes are given by

$$f_G = \frac{G}{G+M}(1 - f_R) \tag{13}$$

and

$$f_M = \frac{M}{G+M}(1 - f_R)\,, \tag{14}$$

where G and M denote the number of PMN and monocytes, respectively.

The series of Bernoulli experiments are described by the Binomial distribution with probability $p_{IC} = 1\,/IC$ to choose an immune cell of type IC, where $IC = G$ and $IC = M$ denotes the number of PMN and monocytes, respectively. The probability of an immune cell of type IC to contain k *C. albicans* cells is then given by

$$p_k^{IC} = \binom{C_{IC}}{k} p_{IC}^k (1-p)^{C_{IC}-k}\,, \tag{15}$$

where C_{IC} denotes the number of *C. albicans* in PMN ($IC = G$) and monocytes ($IC = M$). Due to the fact that for both immune cell types $p_{IC} \ll 1$ and $C_{IC} \gg 1$, the Binomial distribution can be approximated by the Poisson distribution [1]. Thus, the probability for the number of k *C. albicans* cells

within a single immune cell of type IC is given by

$$p_k^{IC} = \frac{\lambda^k}{k!}\, e^{-\lambda}\,. \tag{16}$$

Here, $\lambda = p_{IC} C_{IC}$ represents the mean value of the distribution. The resulting distribution of *C. albicans* cells in PMN and monocytes were compared with the distributions obtained from the SBM simulation by calculating their relative differences (Figure S5).

1. Grinstead CM, Snell JL (1997) Introduction to probability. Providence, RI: American Mathematical Society, 2nd edition.
