# Supplementary material for: A Virtual Infection Model Quantifies Innate Effector Mechanisms and Candida albicans Immune Escape in Human Blood
Source: PLoS Comput Biol. 2014 Feb 20;10(2):e1003479. doi: 10.1371/journal.pcbi.1003479 (PMC3930496; doi:10.1371/journal.pcbi.1003479)
Supplement: Table S1 — Individual start conditions of the fitting algorithm. Start conditions for the parameter fitting algorithm. The number of individuals of alive C. albicans cells in extracellular space (), monocytes () and PMN () at time was stepwise increased by keeping their ratio constant. For each step, the number of runs with corresponding number of fitting steps per run and the range of was adjusted. (PDF) [file pcbi.1003479.s008.pdf]

Supporting Table

Table S1: Individual start conditions of the fitting algorithm.

C(0)	$M_{0,0}(0)$	$G_{0,0}(0)$	number of runs	number of fitting steps	τ
100	50	500	100	3 000	[0.05,5.0]
1 000	500	5 000	100	1 000	[1.0;35.0]
10 000	5 000	50 000	50	500	[1.0;50.0]
100 000	50 000	500 000	50	100	[5.0;80.0]
1 000 000	500 000	5 000 000	50	20	[50.0;100.0]

Start conditions for the parameter fitting algorithm. The number of individuals of alive *C. albicans* cells in extracellular space ($C(0)$), monocytes ($M_{0,0}(0)$) and PMN ($G_{0,0}(0)$) at time $t = 0$ min was stepwise increased by keeping their ratio constant. For each step, the number of runs with corresponding number of fitting steps per run and the range of τ was adjusted.
